# Benchmarking the Nutrition-Related Policies and Commitments of Major Food Companies in Australia, 2018

**DOI:** 10.3390/ijerph17176118

**Published:** 2020-08-22

**Authors:** Gary Sacks, Ella Robinson, Adrian J. Cameron, Lana Vanderlee, Stefanie Vandevijvere, Boyd Swinburn

**Affiliations:** 1 Global Obesity Centre (GLOBE), Institute for Health Transformation, School of Health and Social Development, Deakin University, Geelong, VIC 3220, Australia; ella.robinson@deakin.edu.au (E.R.); adrian.cameron@deakin.edu.au (A.J.C.); 2 School of Nutrition, Université Laval, Quebec, QC G1V 0A6, Canada; lana.vanderlee@fsaa.ulaval.ca; 3 Sciensano, Brussels, 1050 Ixelles, Belgium; stefanie.vandevijvere@sciensano.be; 4 School of Population Health, The University of Auckland, Auckland 1142, New Zealand; boyd.swinburn@auckland.ac.nz

**Keywords:** accountability, commercial determinants of health, food company, obesity, policy, population nutrition

## Abstract

The food industry has an important role to play in efforts to improve population diets. This study aimed to benchmark the comprehensiveness, specificity and transparency of nutrition-related policies and commitments of major food companies in Australia. In 2018, we applied the Business Impact Assessment on Obesity and Population Level Nutrition (BIA-Obesity) tool and process to quantitatively assess company policies across six domains. Thirty-four companies operating in Australia were assessed, including the largest packaged food and non-alcoholic beverage manufacturers (*n* = 19), supermarkets (*n* = 4) and quick-service restaurants (*n* = 11). Publicly available company information was collected, supplemented by information gathered through engagement with company representatives. Sixteen out of 34 companies (47%) engaged with data collection processes. Company scores ranged from 3/100 to 71/100 (median: 40.5/100), with substantial variation by sector, company and domain. This study demonstrated that, while some food companies had made commitments to address population nutrition and obesity-related issues, the overall response from the food industry fell short of global benchmarks of good practice. Future studies should assess both company policies and practices. In the absence of stronger industry action, government regulations, such as mandatory front-of-pack nutrition labelling and restrictions on unhealthy food marketing, are urgently needed.

## 1. Introduction

Unhealthy diets and obesity are the leading contributors to poor health worldwide [1,2]. In Australia, two-thirds of adults and one in four children and adolescents are living with overweight and obesity [3]. The increase in obesity and diet-related non-communicable diseases (NCDs) can largely be attributed to increasingly unhealthy food environments, dominated by the supply, distribution and marketing of packaged, processed foods that are often high in salt, sugar, saturated fat and/or energy [4]. 

As part of a comprehensive societal response to unhealthy diets and obesity, there is global consensus that there needs to be a transition to healthy food environments, in which the foods, beverages and meals that contribute to a healthy diet (as defined by national dietary guidelines) are widely available, affordably priced and widely promoted, and marketing and availability of unhealthy foods is reduced (4). The United Nations (UN) and the World Health Organization (WHO) have recognised the substantial role the food industry plays in contributing to the overall healthiness of food environments, and have made specific recommendations to the food industry of actions they can take [5,6,7,8,9,10]. These include actions (such as reducing the exposure of children (aged < 18) to marketing of unhealthy foods, product reformulation to reduce the levels of salt, sugar, saturated fat and energy content of products, and improved nutrition labelling) that are likely to improve population diets and reduce obesity and diet-related diseases [5,7]. More broadly, the UN Sustainable Development Goals (SDGs) and associated targets present an agenda for all parts of society, including the corporate sector, to work towards improved economic prosperity, and the health and wellbeing of people and the planet [11]. Improving population nutrition represents an important step in achieving the SDGs, with nutrition considered a component of all 17 SDGs [12], and is part of, or linked to, performance targets of several SDG’s including: SDG 2 (zero hunger), SDG 3 (good health and wellbeing), and SDG 12 (responsible consumption and production).

In Australia, there has been only limited assessment of the nutrition-related policies of major food companies. A 2015 assessment of publicly available food company policies on food marketing showed that only 12 (55%) of the largest 22 packaged food manufacturers in Australia had relevant publicly available policies [13]. Existing policies on food marketing to children generally focused on those aged less than 12 years, did not apply to all types of media, and did not provide transparency with respect to the products to which the policies apply. The same 2015 study found that only 13 (59%) of the 22 companies had product reformulation policies, with most of those focused on salt reduction only [13].

Internationally, many large companies and financial investors are now increasingly focusing on monitoring and evaluating their contributions to the SDGs [14]. Moreover, the need for stronger and more comprehensive action to improve the diets of populations has led to a focus on increasing the accountability of major stakeholder groups, including food companies [15]. Currently, there are several initiatives that monitor and assess the policies and actions of the food industry related to nutrition. A prominent example is the Access to Nutrition Initiative (ATNI) that benchmarks large food and beverage manufacturers on their obesity- and undernutrition-related commitments, practices and product portfolios [16,17]. The ATNI has launched three global indexes (2013, 2016, 2018), and a small number of spotlight indices (e.g., in the U.S. [18] and India [19]).

The BIA-Obesity (Business Impact Assessment-Obesity and population-level nutrition) tool and process [20] was developed by INFORMAS (International Network for Food and Obesity/NCDs Research, Monitoring and Action Support)—a global network of public-interest organisations and researchers that monitors public and private sector actions globally [21]—to monitor and benchmark the nutrition-related policies and practices of food companies at the national level. The assessment indicators as part of BIA-Obesity are tailored to different sectors of the food industry, including packaged food and non-alcoholic beverage manufacturers (packaged food and beverage manufacturers), supermarkets and quick service restaurants (also referred to as “fast food restaurants”). Phase 1 of the BIA-Obesity includes a focus on six key policy domains: “corporate strategy”, “product formulation”, “nutrition labelling”, “promotion practices”, “product accessibility”, and “relationships with external groups”. Within each domain, the comprehensiveness, specificity and transparency of company policies and commitments are assessed in relation to global recommendations of best practice [21]. Company policies and commitments score highest when they address all relevant aspects of a particular indicator (e.g., they encompass all marketing channels in relation to reducing the exposure of children to promotion of unhealthy foods), include specific and measurable targets and areas of action, and are publicly available. Phase 2 of the BIA-Obesity assesses company practices in each domain, including the nutritional profile of each company’s product portfolio and their marketing practices. As of August 2020, Phase 1 of BIA-Obesity had been implemented in Australia, New Zealand, Canada and Malaysia [20,22,23,24].

This paper reports the results of the implementation of Phase 1 of the BIA-Obesity in Australia in 2017–2018 (referred to hereafter as *BIA-Obesity Australia 2018*). *BIA-Obesity Australia 2018* aimed to quantitatively assess and benchmark the comprehensiveness, specificity and transparency of the nutrition-related policies and commitments of major food companies in Australia across three sectors: food and beverage manufacturers, supermarkets and quick service restaurants. The objective was to highlight, in an Australian context, where food companies across each sector were demonstrating leadership in obesity prevention and population nutrition in relation to best practice, and make specific recommendations for improvement. The goal was to increase the accountability of food companies for their role in addressing obesity and improving population diets, and stimulate improvements in the healthiness of Australian food environments. 

## 2. Methods

The BIA-Obesity methods have previously been described [20]. Briefly, the process of implementing the BIA-Obesity in a particular country involves: (1) tailoring the BIA-Obesity to the local context; (2) selecting companies for inclusion in the assessment; (3) collecting publicly available data on company policies and commitments; (4) engaging with company representatives to verify and supplement publicly available data; (5) scoring each company’s policies and commitments using the country-specific BIA-Obesity tool; (6) developing recommendations for each company and sector; and (7) reporting results. An overview of the way in which this process was applied as part of *BIA-Obesity Australia 2018* is provided below. Throughout the study, the term “healthy” foods was used to refer to foods and drinks (such as fruit, vegetables, wholegrain cereals, lean meats, and reduced fat dairy) recommended for daily consumption in the Australian Dietary Guidelines [25]. “Unhealthy” foods was used to refer to energy-dense, nutrient-poor foods and drinks (such as foods containing added sugars and salt, and foods high in saturated fat) that, according to the Australian Dietary Guidelines, should be consumed only sometimes and in small amounts [25]. Ethics for this study was granted by Deakin University HEAG-H, project ID: HEAG-H 205_2016.

### 2.1. Adaptation of the BIA-Obesity for the Australian Context

The global methodology for the BIA-Obesity was tailored to the Australian context through consultation with experts within the INFORMAS network and in light of local knowledge of the food environment and the regulatory context in Australia. Specifically, the indicators and scoring criteria within each of the six domains of the BIA-Obesity were adapted to suit the Australian packaged food and beverage manufacturing, supermarket and quick service restaurant sectors. In the “product formulation” domain, we included indicators related to company engagement with the Healthy Food Partnership (a public–private partnership between the Australian government, food industry and public health nutrition groups) [26]. In the “nutrition labelling” domain, the global indicators from the BIA-Obesity tool were adjusted to reflect the regulatory requirements for food labelling that apply in Australia. For example, indicators related to the provision of general nutrition information on the back-of-pack were removed as regulations in this area were mandatory, and, therefore, voluntary commitments were not relevant. Company commitments to label trans fat and indicate kilojoule content on menus (for quick service restaurants) were retained as they were not mandatory requirements in all jurisdictions in Australia. Indicators were added to include an assessment of company commitment to implementation of the Australian government-endorsed Health Star Rating (HSR) front-of-pack labelling system [27]. In several domains (“product formulation”, “nutrition labelling”, “promotion practices”, “product accessibility”) company use of classification systems for defining the relative healthiness of products was given a higher score if they aligned with Australian government-endorsed guidelines, such as The Australian Dietary Guidelines [25] or the nutrient-profiling scoring criteria underpinning health claims regulations [28]. The relative weighting of each domain (specific to each sector) was retained from the weighting specified in the global methodology for BIA-Obesity [20]. The assessment tool, including domains, indicators, scoring and weighting for *BIA-Obesity Australia 2018* is available in the Supplementary Material, Table S1 (packaged food and beverage manufacturers), Table S2 (supermarkets), Table S3 (quick service restaurants).

### 2.2. Selection of Companies

The largest food companies operating in Australia were selected based on market share in four sectors (packaged food manufacturers, non-alcoholic beverage manufacturers, supermarkets and quick-service restaurants), using 2017 data from the Euromonitor *Passport* database [29]. In line with the approach recommended by INFORMAS as part of the BIA-Obesity methods [20], we aimed to select companies that accounted for approximately 80% of the market share in each of the supermarket and quick service restaurant sectors, and at least 50% of the market share in each of the packaged food and non-alcoholic beverage manufacturing sectors. While the largest supermarket retailers in Australia were classified by Euromonitor as amongst the largest food and beverage manufacturers, due to the relatively large market share of their own-brand products, they were assessed using the supermarket-specific version of the *BIA-Obesity Australia 2018* tool. In some cases, the corporate entities selected were adjusted to the country context to account for the level at which company policy decisions were made and reported (e.g., *KFC* and *Pizza Hut*, both owned by *Yum! Brands Inc*, were assessed separately; *Chicken Treat*, *Oporto* and *Red Rooster,* all owned by *Quick Service Restaurant Holdings Pty Ltd*, were assessed separately; *Restaurant Brands International Inc* was assessed as *Hungry Jack’s*; *Doctor’s Associates Inc* was assessed as *Subway*; *Lion Pty Ltd* was assessed as *Lion Dairy and Drinks*; *Smiths Snackfoods Co* was assessed as *PepsiCo*; *Wesfarmers Ltd* was assessed as *Coles*).

### 2.3. Collection ofPpublicly Available Information

We ran a systematic process (between February to December 2017) to collect publicly available information on company policies and commitments related to obesity prevention and population nutrition. This included searches of company websites (national, global and brand-specific, where relevant) and company reports (e.g., corporate responsibility and sustainability reports). Websites of relevant industry associations and government departments were also searched to locate applicable policies, industry memberships and policy positions. Relevant webpage and document information in relation to each *BIA-Obesity Australia 2018* indicator were saved and copied into an Excel spreadsheet. This information was then used to pre-populate a survey that could be sent to company representatives for review.

### 2.4. Company Engagement 

In June 2017, we invited companies to participate in the project. Contact information (phone numbers and/or email addresses) of senior company representatives in nutrition and wellbeing, corporate social responsibility and/or corporate affairs roles were identified through known contacts of the research team and their collaborators (e.g., in public health organisations in Australia) as well as searches of company reports, corporate websites, media releases and social media content. In some cases, contact details for specific individuals were not able to be identified and, as such, an introductory email was sent to a generic company email address (e.g., customer service, general enquiry). Introductory emails outlined the rationale for the project, provided a project summary, and requested that the company nominate a representative who could liaise with the project team throughout the duration of the project. Where companies nominated a representative and they agreed to participate in data collection processes (“participating companies”), they were sent a plain language statement and consent form to complete. The publicly available information that had been collected was then relayed to company representatives to validate and supplement. Companies were given the opportunity to sign non-disclosure agreements if requested. These agreements stipulated that confidential information provided by the company to the research team would be used for assessment purposes only, and the detailed content would not be made publicly available. The process of engaging with companies to supplement publicly available data involved multiple conversations and follow-up requests by email and phone, with the level of engagement varying by company based on their stated requirements. For those companies that did not respond or declined to participate in data collection processes, the assessment was based on publicly available information only, and the companies were informed of this.

In November 2017, all companies included in the study were invited to attend a workshop to discuss preliminary findings. In a limited number of cases, companies supplied additional information regarding their policies and commitments subsequent to the workshop that was used as part of the final assessment. Only policies and commitments up to 31 December 2017 were eligible for inclusion.

### 2.5. Data Analysis and Scoring Companies Against Domains and Indicators of the Tool

Two researchers (E.R. and G.S.), both of whom were familiar with the BIA-Obesity methods, independently analysed policy information for each company and scored companies against the indicators of the tool, with each domain receiving a pre-specified weighting [20] to derive an overall score out of 100 for each company. In addition, each company’s individual domain scores were converted to a score out of 100 for domain-specific comparison purposes. Indicators that were not relevant to certain companies, due to the nature of their product portfolio or nutrition-related activities, were marked as “not applicable” and the total possible scores by domain and overall were adjusted accordingly. For example, in the “product formulation” domain, indicators related to sodium, saturated fat and trans fat were considered as “not applicable” for companies that produced only sugar-sweetened beverages (not including milk-based beverages). In the “relationships with external groups” domain, if the company explicitly stated they had no relevant external relationships, this domain was marked as “not applicable”. Discrepancies in scoring between assessors were resolved through in-depth discussion within the research team. The reliability of the tool was assessed by examining inter-rater reliability for a sample of companies. The Gwet’s AC1 (unweighted) interrater reliability coefficients (calculated using Agreestat 2015.6.1 software, Advanced Analytics, Gaithersburg, MD, USA) were 0.98 (95% confidence interval [CI]: 0.96–0.99). 

### 2.6. Development of Recommended Actions

Recommended actions were developed and prioritised by the research team based on WHO guidelines (Global Strategy on Diet, Physical Activity and Health [5], Global Action Plan for the prevention and control of non-communicable diseases 2013–2020 [7], and Report of the Commission on Ending Childhood Obesity [8]), the assessment criteria of the *BIA-Obesity Australia 2018* tool, and international benchmarks of “best available” company performance in the area (refer to Supplementary Material, Table S4 for examples of good practice in each domain). Where relevant, recommendations were aligned with the goals of the Healthy Food Partnership, through engagement with members of the Food Service Working Group within the Healthy Food Partnership.

At the industry stakeholder workshop in November 2017, the research team sought feedback on recommendations at a sector level. Feedback during and after the workshop was used to tailor sector-level recommendations to be specific to each company so that their relevance to that particular company was maximised. For example, where the sector-level recommendation stated, “*Eliminate* use of promotion techniques (e.g., cartoon characters, interactive games) with strong appeal to children in relation to “less healthy products and brands”, for a particular food manufacturer, the recommendation was changed to, “*Extend commitment* to eliminate use of promotion techniques with strong appeal to children in relation to ‘less healthy’ products and brands, by ensuring commitment applies also to product packaging”. 

### 2.7. Release of Results

Results from the *BIA-Obesity Australia 2018* assessment were synthesized and collated into a report for each sector. Each report included overall assessment of each company in the sector, analysis by domain and individual company scorecards. Recommendations were also provided at the sector- and company-level (overall and by domain), including international and national examples of good practice. Three reports were released: “Inside our supermarkets” (February 2018) [30], “Inside our food and beverage manufacturers” (March 2018) [31] and “Inside our quick service restaurants” (May 2018) [32]. Two weeks prior to the public release of each report, an embargoed copy of the report and media release was shared with each relevant company.

## 3. Results

### 3.1. Companies Included in the Assessment

In total, 19 packaged food manufacturers (representing 56.5% of the total market share), six non-alcoholic beverage manufacturers (72.8% of the total market share), four supermarkets (76.7% of the total market share), and 11 quick service restaurants (67.1% of the total market share) were identified for inclusion in the assessment. This corresponded to a total of 34 companies, with three of the packaged food manufacturers (*Aldi*, *Woolworths*, *Coles*) included in the supermarket sample, and three of the beverage manufacturers (*Lion Dairy and Drinks*, *Kraft Heinz*, *Parmalat*) already included in the packaged food manufacturers sample due to their diverse product portfolio (i.e., both food and beverages). Companies selected for inclusion in the *BIA-Obesity Australia 2018* assessment and their corresponding market share are listed in Table 1. The majority of companies were headquartered internationally (59%), followed by Australia (35%), Australia and New Zealand (3%), and New Zealand (3%). The major product categories of each company varied. For packaged food manufacturers, common product categories included “dairy”, “bread and bakery”, “cereal and grain” and “sauces, dressings, spreads and dips”. For supermarkets (own-brand products only) common product categories included “bread and bakery”, “fruit and vegetables” and “dairy”. For quick service restaurants, the dominant product portfolio was either “chicken” or “burgers”.

**Figure 1:**
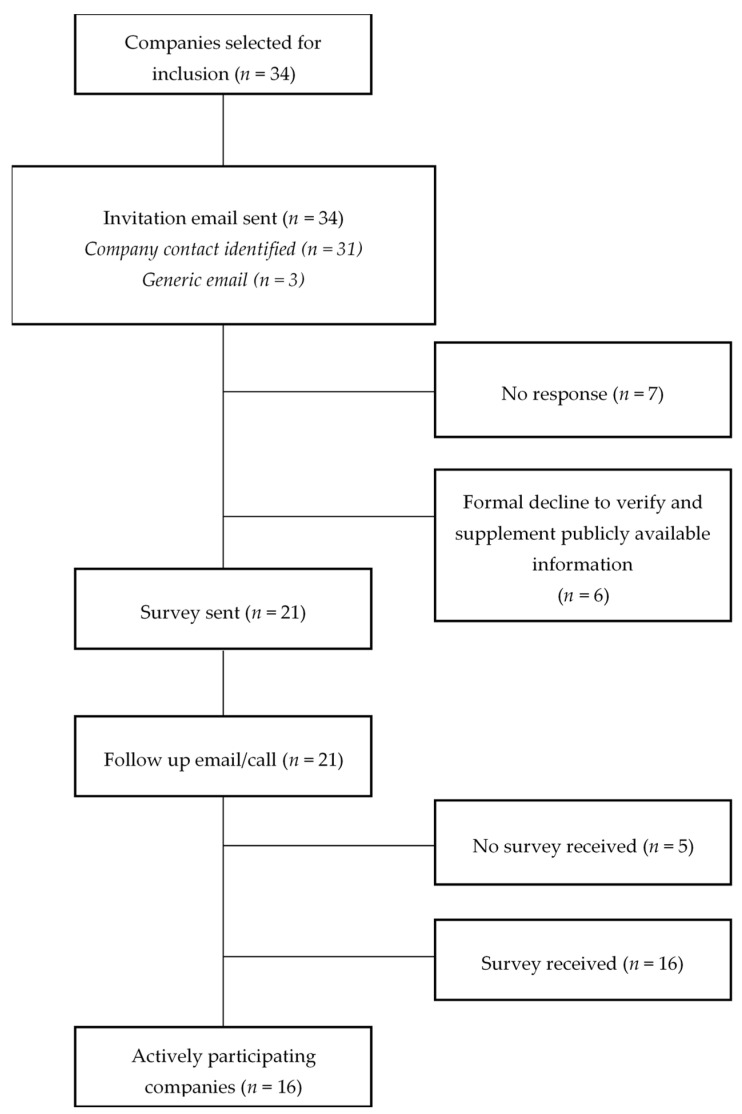
Participant flow diagram showing the levels of engagement of the companies included in *BIA-Obesity Australia 2018*.

Sixteeen out of 34 companies (47%) actively participated in data collection processes by agreeing to verify and supplement publicly available information. The extent of participation varied by sector: 11 out of 19 (58%) food and beverage manufacturers; 3 out of 4 (75%) supermarkets; and 2 out of 11 (18%) quick service restaurants participated in data collection processes. Two of the “participating” companies requested that the research team sign non-disclosure agreements. For the remaining companies (*n* = 18), the assessment was based on publicly available information only. Six companies expressly declined to verify and supplement publicly available data. For seven companies, no response was ever received from the company, despite multiple attempts to make contact. In most of these cases no appropriate company contact person was identified during the company engagement process. Refer to Figure 1 for the participant flow diagram, which indicates levels of engagement of companies included in *BIA-Obesity Australia 2018*.

### 3.2. Company Performance 

**Figure 2:**
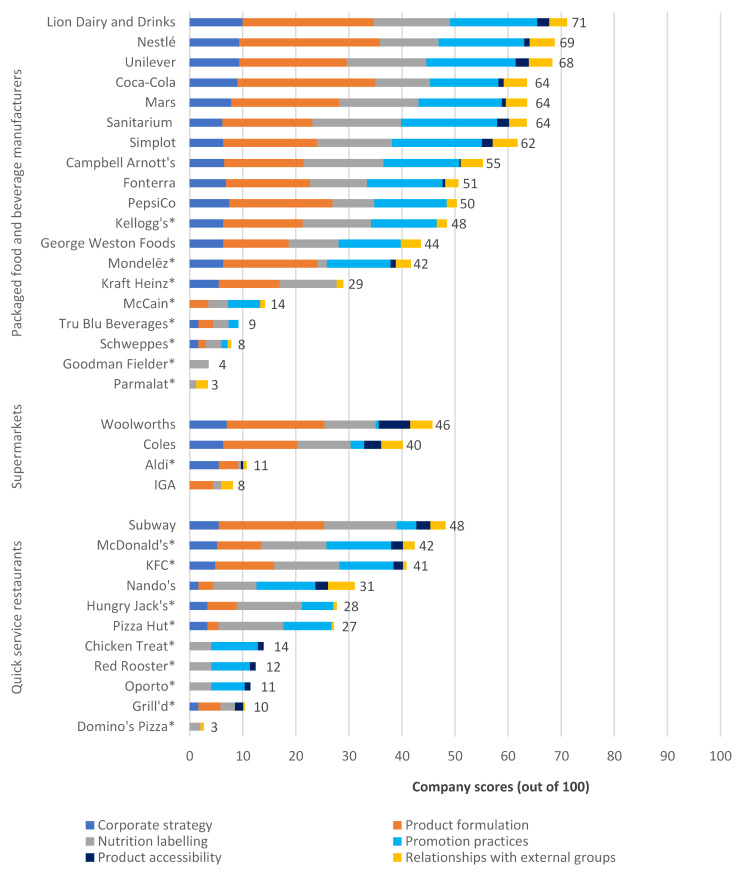
*BIA-Obesity Australia 2018,* company scores by sector (out of 100); * Assessment based on publicly available information only.

All companies demonstrated some stated commitment to addressing health and nutrition, with scores ranging from 3/100 to 71/100 (median: 40.5/100; IQR: 39.5). The packaged food and beverage manufacturing sector was the highest performing sector overall (median: 50/100; range: 3/100–71/100; IQR: 42.5). Few companies in the supermarket (median: 25.5/100; range: 8/100–46/100; IQR: 31.25) and quick service restaurant (median: 27/100; range: 3/100–48/100; IQR: 24.5) sectors had comprehensive and specific policies and commitments in place. Details of company scores across each of the policy domains are shown in Figure 2 and Table 2.

#### 3.2.1. Corporate Strategy 

The “corporate strategy” domain assesses overarching company policies and commitments to addressing obesity and improving population-level nutrition. This was the highest performing domain overall, with a median score of 55/100 (range: 0–100; IQR = 48) across all sectors. Leading companies included *Lion Dairy and Drinks*, *Nestlé* and *Unilever*. Companies in the packaged food and beverage manufacturing sector performed better than companies in other sectors, with top performers having corporate strategies that included a commitment to address nutrition and health-related issues and routine publicly available reporting against their approach to achieving nutrition and health targets. Top performers from the food and beverage manufacturing sector, along with *Woolworths* in the supermarket sector, aligned their nutrition and health approach with the UN SDGs and/or priorities set out by the WHO. In the quick service restaurant sector, companies had fewer strategic commitments to addressing nutrition and health-related issues, and only reported limited detail of their nutrition and health approach publicly. Across all sectors, the lowest performing companies made little or no mention of nutrition or obesity-related issues as part of corporate reporting. 

#### 3.2.2. Product Formulation

The “product formulation” domain assesses company policies and commitments regarding product development and reformulation to reduce nutrients of concern (i.e., sodium, free sugars, saturated fat, trans fat) and energy content. The median score was 40/100 (range: 0–88; IQR = 49) across all sectors. Leading companies included *Nestlé*, *Coca-Cola* and *Lion Dairy and Drinks*. A total of 17 out of 19 packaged food and beverage manufacturers, three out of four supermarkets, and six out of 11 quick service restaurants reported having taken some action to reduce one or more nutrients of concern in products/menu items. The most common area where companies had reported taking some action to reduce a nutrient of concern was sodium (*n* = 21/31), followed by sugars (*n* = 22/34), saturated fat and trans fat (*n* = 18/31). Of the areas assessed, companies had the fewest commitments in relation to portion size/serving size (*n* = 12/34). In the packaged food and beverage manufacturing sector, several companies (including *Nestlé*, *Lion Dairy and Drinks*, *Mars* and *Unilever*) had a commitment to not use any artificially produced trans fat in products, and/or had reported complete removal of trans fat across their portfolios.

A number of company reformulation commitments were vague or unpublished, with very few measurable and specific targets in place. Unlike top performers in the packaged food and beverage manufacturing sector, companies in the supermarket and quick service restaurant sector had limited or no routine reporting against progress in achieving specific reformulation targets.

A number of companies were participants in the Australian Government’s Healthy Food Partnership, including *Nestlé*, *Coca-Cola*, *Lion Diary and Drinks*, *Simplot*, *Sanitarium*, *Fonterra*, *PepsiCo*, *Campbell Arnott’s*, *Woolworths*, *Coles*, *Subway* and *KFC*. 

#### 3.2.3. Nutrition Labelling

The “nutrition labelling” domain assesses company policies and commitments regarding the disclosure and presentation of nutrition information on product packaging, online and on menus (where relevant). The median score was 54/100 (range: 3–91; IQR = 56) across all sectors.

Thirteen out of 23 companies in the packaged food and beverage manufacturing and supermarket sectors had publicly committed to implement the HSR front-of-pack labelling system across all or some of their product portfolios. Notably, the two largest supermarkets, *Woolworths* and *Coles*, had committed to implementation of HSR labelling on their full product range. Several other packaged food and beverage manufacturers had only committed to implement the HSR system across certain relevant categories of their portfolio. *Coles* was also notable for having in place a clear system for determining whether nutrition claims (on own-brand products) could be placed on products, based on the healthiness of the product. No company publicly committed to routinely label added sugars or artificially produced trans fat on their products.

In the quick service restaurant sector, six companies committed to implement kilojoule menu board labelling across all states and territories (nationwide). At the time the *BIA-Obesity Australia 2018* study was conducted, it was a legislative requirement for quick service restaurants to display kilojoule content on menu boards in all states and territories except Tasmania, Western Australia and the Northern Territory.

All companies in the packaged food and beverage manufacturing and quick service restaurant sectors and two companies in the supermarket sector provided online nutrition information for all or some products/menu items. 

#### 3.2.4. Promotion Practices

The “promotion practices” domain assesses company policies and commitments for reducing the exposure of children (aged < 18) and adults to promotion of unhealthy foods and brands. This domain was one of the lowest performing domains, with a median score of 36/100 (range: 0–60; IQR = 42) across all sectors.

Thirteen packaged food and beverage manufacturers and seven quick service restaurants had signed on to voluntary ‘responsible’ children’s marketing codes, through the Australian Food and Grocery Council (AFGC) industry association.

Companies with a commitment in the area specified that they would not directly target children under the age of 12 years (packaged food and beverage manufacturers) or 14 years (quick service restaurants). The majority of packaged food and beverage manufacturers with a commitment in the area defined the audience for which marketing restrictions would apply as either “where the audience is predominantly children under 12” or “where 35% or more of the audience is children under 12”. *Mars* had the most stringent definition of “child” audience for the purposes of marketing restrictions, with their policy committing to avoid promotion of unhealthy products “where 25% or more of the audience is under 12”. *Kellogg’s* and *PepsiCo* adopted the least stringent definition of “child” audience, with their policies stating that they would reduce promotion of unhealthy products “where 50% or more of the audience is under 12”.

Across all companies, no commitments explicitly restricted all times/events when a large number of children were likely to be exposed. Several companies, including *Coca-Cola*, *KFC*, *Mars*, *Mondelēz*, *Nestlé*, *Pizza Hut* and *Unilever* specifically stated that their marketing policy applied to all forms of media or marketing communications. Other companies with a commitment in the area generally specified that their policy applied only to television, radio, print, cinema and third-party internet sites; however, many packaged food and beverage manufacturers and quick service restaurants did not provide details on the media to which their policies applied.

Several packaged food and beverage manufacturers (including *Sanitarium*, *Simplot*, *Lion Dairy and Drinks* and *Unilever*) used Australian government-endorsed guidelines to classify the healthiness of products for the purposes of marketing to children, whilst other companies relied on their own nutrient-profiling systems.

No companies in the supermarket sector had developed formal policies that would effectively restrict the exposure of children to promotion of “less healthy” products, or restrict the promotion of “less healthy” foods in-store or in their promotional catalogues. Unlike other sectors of the food industry (packaged food and beverage manufacturers and quick service restaurants), there was no available sector-level voluntary marketing code for supermarkets. 

#### 3.2.5. Product Accessibility

The “product accessibility” domain assesses company policies and commitments to restrict the availability and affordability of unhealthy products and improve the affordability and availability of healthy foods. Across all sectors, companies had limited commitments in the area, and “product accessibility” was the lowest scoring domain overall, with a median score of 5/100 (range: 0–50; IQR = 16).

The highest performers in this domain were *Unilever*, *Lion Dairy and Drinks* and *Sanitarium*. The scores varied substantially between the sectors, with the highest performing packaged food and beverage manufacturer (*Unilever*) scoring 50/100, compared to the highest scoring quick service restaurants (*Subway* and *Nando’s*) scoring 13/100. In the packaged food and beverage manufacturing sector, *Unilever* clearly identified availability and affordability of healthy products as a key part of its business strategy, while *Lion Dairy and Drinks* publicly committed to work with retailers to position healthier products at the front of store, where they are more accessible. In the supermarket sector, *Woolworths* was the only company to commit to providing some confectionery-free checkouts in its stores (one in the majority of stores nationally), whilst no companies had a commitment to make all checkouts confectionery-free in their stores. *Subway* was the only quick service restaurant to commit to providing both a healthier side and drink option as the default option in all children’s meals. No companies publicly supported fiscal policies to make healthier foods cheaper and unhealthy foods relatively more expensive. *Coca-Cola* disclosed their position on sugar sweetened beverage taxation, stating that they opposed “discriminatory soft drink taxes”. 

#### 3.2.6. Relationships with External Groups

The “relationships with external groups” domain assesses company policies and commitments with respect to company engagement and support provided to external organizations (e.g., governments, political parties, professional associations, research organizations, and community groups) related to health and nutrition. The median score for this domain was 44/100 (range: 0–94; IQR = 67) across all sectors. Top performers in the packaged food and beverage manufacturing and supermarket sectors declared most types of relevant external relationships and their support for research (if any). *Coca-Cola* was the only company to disclose, at a national level, funding amounts provided to external groups, including for research, and updated this information on a regular basis. In the quick service restaurant sector, companies had limited disclosure of their engagement with external groups related to nutrition and health. Across all sectors, company support for charitable groups and charity initiatives (often referred to as “corporate philanthropy”) was the aspect of this domain that was most consistently reported.

Several companies (*Campbell Arnott’s*, *George Weston Foods*, *Mars*, *Nestlé*, *Unilever*, *Coles*) had formal published policies that prohibited political donations. *Woolworths* disclosed all political donations annually in its sustainability report. Four other companies had an internal commitment (not publicly disclosed) to not give political donations. 

### 3.3. Recommended Actions for Companies

The set of recommended actions for each sector, prepared as part of the study, are outlined in Table 3. In each domain, recommendations broadly highlight the need for nutrition-related policies and commitments that are specific, comprehensive, transparent and nationally applicable. Individual company scorecards are available in Supplementary Material, File S1.

## 4. Discussion

The *BIA-Obesity Australia 2018* assessment was the first of its kind to benchmark the comprehensiveness, specificity and transparency of nutrition-related policies and commitments of major food companies in Australia across three sectors (packaged food and beverage manufacturers, supermarkets and quick service restaurants). The assessment demonstrated that, while Australian food companies had acknowledged the role they can play in this important area of public health, there was substantial room for improvement in each company. 

The study identified that companies were taking several positive steps, including acknowledging nutrition and health issues as part of corporate reporting (26 out of 34 companies), committing to reduce levels of nutrients of concern (26 out of 34 companies), publicly committing to implement the Australian government’s Health Star Rating food labelling scheme (14 out of 23 companies for which this was relevant), and committing to implement kilojoule menu board labelling nationwide (6 out of 11 companies for which this was relevant). However, in other areas, such as “product accessibility” and “promotion practices”, the commitments that companies had made fell short of best practice recommendations. On average, the packaged food and beverage manufacturers included in the study performed better than the supermarkets and quick service restaurants. Supermarkets scored particularly low in the “promotion practices” domain, in reflection of their limited policies and commitments in the area. In comparison to packaged food and beverage manufacturers, quick service restaurants scored substantially lower overall in relation to “corporate strategy”, “product formulation”, and “relationships with external groups”. Within each sector, company scores varied substantially, indicating important differences in the extent to which companies had committed to addressing nutrition-related issues. There was no discernable pattern to the characteristics of companies that performed well. As examples, amongst the top performers, there was a mix of Australian-based companies and companies with headquarters located internationally, and, in respect of major product categories, *Lion Dairy and Drinks* (dairy products) were the highest scoring company (71/100), whereas *Parmalat* (dairy products) only scored 3/100. Detailed investigation of the drivers (both internal and external) for companies to take action, and leverage points for change, is required.

The results of this study were consistent with previous assessments (conducted in 2015) of food and beverage manufacturer nutrition policies in Australia. The 2015 study showed that, across the board, company policies on food marketing and product reformulation fell far short of global recommendations [13]. The results of this study were also consistent with assessments of food and beverage manufacturers conducted as part of the ATNI, which have found large variation in the policies and commitments of major food and beverage manufacturers at the global level [16]. In the ATNI 2018 Global Index, company scores varied between 0/10 and 6.8/10 across the largest food and beverage manufacturers [16]. The highest scoring domain in the ATNI 2018 Global Index was “governance” and the lowest scoring domain was “accessibility” [16]. These findings are in line with the scoring from *BIA-Obesity Australia 2018*, where the range of scores was similar (3/100 to 71/100), and the highest and lowest scoring domains (“corporate strategy” and “product accessibility”, respectively) were equivalent. The findings from *BIA-Obesity Australia 2018* are also in line with the findings from BIA-Obesity assessments conducted in New Zealand in 2018 [22]. In BIA-Obesity New Zealand, the overall median score for all sectors was 38/100 (compared to 40.5/100 for Australia), and the median score for the packaged food and beverage manufacturing sector was 47/100 (compared to 50/100 for Australia). However, the scores observed in Australia were higher than those observed in BIA-Obesity Canada (2018) (median score for packaged food and beverage manufacturers = 27/100) [23] and substantially higher than those observed in BIA-Obesity Malaysia (2019) (median score for all sectors = 11/100) [24]. Furthermore, where particular companies were assessed in Malaysia, Australia and New Zealand, those companies were found to score lower overall in BIA-Obesity Malaysia [24]. The level of variation in BIA-Obesity scores achieved by companies across countries indicates that there was substantial variation in the comprehensiveness and specificity of company policies and commitments at the country level, and the extent to which these are disclosed by companies. This is likely to reflect differences in each country’s social and regulatory context, including political and economic factors that may influence food company policy and practice [13].

With regard to supermarkets, a 2018 study examining corporate social responsibility reporting of the largest 100 supermarkets globally found that whilst supermarkets had taken action to report on sustainability-related issues, there was a limited focus on nutrition and health [35]. This aligns with the findings of *BIA-Obesity Australia 2018*, where supermarkets had limited disclosure around nutrition-related policies and commitments and scored low overall. None of the supermarkets included in this study had policies in place to restrict the promotion of unhealthy foods and/or increase the promotion of healthy foods in-store (e.g., at end-of-aisle displays and island bins) or in their promotional catalogues. This is concerning given that Australian supermarkets have been shown to heavily promote unhealthy items in prominent in-store locations [36] and in their catalogues [37]. Supermarkets in Australia were also found to be lagging when compared to supermarkets in other countries. For instance, in the United Kingdom, the majority of major retailers had pledged to remove confectionery from all checkouts [38]; however, no supermarkets in Australia had made such commitments. 

Overall, 16 of the 34 companies (47%) included in *BIA-Obesity Australia 2018* engaged with the research team as part of data collection processes. Participation rates were very similar between BIA-Obesity studies conducted in 2018 in Australia (47%), New Zealand (48%) and Canada (50%); however, they were substantially higher than participation rates in Malaysia (18%) [23,24,25]. In this study, there was a higher level of engagement from packaged food and beverage manufacturers and supermarkets compared to quick service restaurants. Companies that participated in the data collection process scored substantially better overall, with a mean score of 52/100 for “participating” companies, compared to a mean score of 19/100 for non-participating companies. In most cases, the higher scores of “participating” companies reflected their pre-existing policy focus on nutrition and health (e.g., *Lion Dairy and Drinks*, *Nestlé*, *Mars*), rather than their disclosure of additional policy information to the research team as part of engagement processes. Nevertheless, engagement with “participating” companies did reveal important internal policy information in several instances. This reflects the need for increased transparency and public disclosure across the board. While no companies committed to policy change as part of the *BIA-Obesity Australia 2018* engagement process, an evaluation of the impact of *BIA-Obesity Australia 2018* indicated that greater company engagement with the benchmarking process increased the likelihood that the study would lead to policy and practice change within each company [39]. 

Current Australian government policy to improve the healthiness of food environments relies heavily on voluntary industry actions through initiatives such as the HSR nutrition labelling scheme, the Healthy Food Partnership, and voluntary industry codes in the area of food marketing to children. While many of the packaged food and beverage manufacturers and supermarkets included in *BIA-Obesity Australia 2018* had committed to implement the HSR scheme, it is likely that greater incentives are required for additional companies to voluntarily adopt the scheme. A number of packaged food and beverage manufacturers and supermarkets included in this study were participants in the Healthy Food Partnership [40]. While this provides some indication that food companies were willing to work with government to address health and nutrition issues, the scope of the Healthy Food Partnership is limited, and it is unclear how progress will be systematically monitored or how companies would be held accountable for taking actions as part of the initiative [41]. Initiatives similar to the Healthy Food Partnership in other countries, for example the United Kingdom Public Health Responsibility Deal, have faced criticism for limited voluntary action, poorly implemented monitoring and evaluation processes, and a lack of sanctions for companies failing to meet targets [42,43]. With regard to voluntary industry codes related to food marketing, several Australian and global studies have indicated their failure to protect children from exposure to promotions for unhealthy food [44,45].

In light of the results of *BIA-Obesity Australia 2018* and the limitations of relying on voluntary industry actions, governments in Australia need to implement stronger policy interventions, such as mandatory implementation of the HSR labelling system, mandatory nutrient-specific limits and targets by food category, and comprehensive mandatory restrictions on unhealthy food marketing. These types of interventions have recently been implemented in several countries. For example, Chile has implemented mandatory black warning labels on food products that exceed limits for sugar, salt, saturated fat and energy content, coupled with comprehensive restrictions on promotion of these products [46]. Evaluation of Chile’s laws have demonstrated the effectiveness of this approach [47]. Mexico has also implemented similar warning labels to Chile [48]. Several countries have mandated the elimination of industrially produced trans fat [49], and a number of countries, including Argentina and South Africa, have placed mandatory limits on sodium content in certain food categories [50]. 

A key strength of this study is that we applied a standardized global tool and process, tailored to the local context, with assessment criteria developed based on best practice public health recommendations. In addition, we undertook detailed engagement with companies as part of the benchmarking process, thereby increasing the comprehensiveness and accuracy of data collected, and potentially improving the likelihood that the recommendations would lead to company-level change. 

An important limitation of the study was that not all companies engaged in the data collection process. Accordingly, assessment of some companies was based on publicly available information only. As with the experience of ATNI, we expect to have increased engagement from companies in future assessments. This research represented only Phase 1 of the BIA-Obesity, which assesses company policies and commitments, but does not take into account the extent to which these commitments are implemented in practice. While the development and disclosure of policies is likely to lay the foundation for good practice and provides an opportunity to encourage accountability, policies and commitments do not necessarily lead to changes in practices. Importantly, the company scores from this study are not intended to reflect the ‘healthiness’ of a company, particularly because the assessment did not take into account the healthiness of the company’s product portfolio. Future studies in Australia should be conducted to investigate changes in company policies and commitments over time. Such studies could also explore the contribution of accountability mechanisms, such as *BIA-Obesity Australia 2018*, to change. In addition, future studies should investigate the extent to which policies and commitments translate into practice, including assessment of the healthiness of the company’s product portfolio, and the extent and nature of food marketing. Finally, the study focused only on selected aspects of food company nutrition policies and commitments. The study did not assess other aspects of nutrition-related policy, e.g., marketing of breastmilk substitutes and labelling of fibre content, as well as wider issues, such as environmental sustainability, that are of public concern [51]. This should be the subject of future studies.

## 5. Conclusions

This 2018 study demonstrated that major food companies in Australia had taken some steps to address population nutrition and obesity-related issues. However, across all sectors, there was large-scale variation amongst companies and substantial room for improvement. Overall, the observed policies and commitments from the food industry were likely to be insufficient to meaningfully address population nutrition issues in Australia. Accordingly, governments need to closely monitor how the policies and commitments of food companies change over time, the extent to which they are implemented, and the healthiness of Australian food environments. Where governments rely on voluntary actions from food companies, greater support and incentives for food companies are likely to be required, along with sanctions for lack of action. Other stakeholder groups, including public health organisations, researchers and investors, also need to monitor company progress, particularly as part of evaluation of company contributions to the UN Sustainable Development Goals. This research provided a critical first step in monitoring food industry nutrition-related action in Australia and establishing accountability mechanisms. In areas where voluntary company actions were found to be insufficient, governments need to urgently implement stronger policies, such as elimination of industrially produced trans fats, mandatory front-of-pack nutrition labelling and restrictions on unhealthy food marketing.

## Figures and Tables

**Table 1:** Companies selected for inclusion in the *BIA-Obesity Australia 2018* assessment.

Company	Country of Headquarters	Major Product Categories ^1^	Market Share (Sector Rank) ^2^	Agreed to Verify and Supplement Publicly Available Information (yes/no)
**Packaged food manufacturers**				
Aldi Stores Supermarkets Pty Ltd (Aldi) ^3^	Germany	Dairy; Bread and bakery products; Fruit and vegetables	8% (1)	No
Woolworths Ltd (Woolworths) ^3^	Australia	Fruit and vegetables; Bread and bakery products; Convenience foods	6.2% (2)	Yes
Wesfarmers Ltd (Coles) ^3^	Australia	Bread and bakery products; Fruit and vegetables; Meat and meat products	5.5% (3)	Yes
Lion Pty Ltd (Lion Dairy and Drinks) ^4^	Australia	Dairy; Non-alcoholic beverages	4.4% (4)	Yes
Mondelēz Australia Pty Ltd (Mondelēz)	United States	Confectionery; Bread and bakery products; Dairy	4.1% (5)	No
Parmalat Australia Pty Ltd (Parmalat)	Italy	Dairy	3.4% (6)	No
Simplot Australia Pty Ltd (Simplot)	United States	Fish and fish products; fruit and vegetables; Sauces, dressings, spreads and dips	2.7% (7)	Yes
Nestlé Australia Ltd (Nestlé)	Switzerland	Cereal and grain products; Confectionery; Non-alcoholic beverages	2.7% (8)	Yes
Arnott’s Biscuits Ltd (Campbell Arnott’s)	United States	Bread and bakery products; Convenience foods; Non-alcoholic beverages	2.6% (9)	Yes
Goodman Fielder Ltd (Goodman Fielder)	Australia	Bread and bakery products; Sauces, dressings, spreads and dips; Cereal and grain products	2.2% (10)	No
Unilever Australia Ltd (Unilever)	The Netherlands/ United Kingdom	Dairy; Convenience foods; Sauces, dressings, spreads and dips	2.1% (11)	Yes
Mars Australia Pty Ltd (Mars)	United States	Sauces, dressings, spreads and dips; Confectionery; Cereal and grain products	1.9% (12)	Yes
Fonterra Brands (Au) Pty Ltd (Fonterra)	New Zealand	Dairy; Edible oils and oil emulsions; Snack foods	1.9% (13)	Yes
Smith’s Snackfoods Co (PepsiCo) ^5^	United States	Snack foods; Sauces, dressings, spreads and dips; Bread and bakery products	1.8% (14)	Yes
George Weston Foods Ltd (George Weston Foods)	Australia	Bread and bakery products; Meat and meat products	1.8% (15)	Yes
McCain Foods Pty Ltd (McCain)	Canada	Convenience foods; Fruit and vegetables; Bread and bakery products	1.4% (16)	No
Heinz Co Australia Ltd (Kraft Heinz)	United States	Fruit and vegetables; Convenience foods; Non-alcoholic beverages	1.4% (17)	No
Kellogg Pty Ltd (Kellogg’s)	United States	Cereal and grain products; Special foods	1.3% (18)	No
Sanitarium health food co (Sanitarium)	Australia and New Zealand	Cereal and grain products; Dairy; Special foods	1.1% (19)	Yes
**Total**			56.5%	
**Non-alcoholic beverage manufacturers**				
Coca-Cola Amatil Ltd (Coca-Cola)	United States	Non-alcoholic beverages	31.1% (1)	Yes
Schweppes Australia Pty Ltd (Schweppes)	United States	Non-alcoholic beverages	23.7% (2)	No
Lion Pty Ltd (Lion Dairy and Drinks) ^6^	Australia	Dairy; Non-alcoholic beverages	8.3% (3)	Yes
Heinz Co Australia Ltd HJ (Kraft Heinz) ^6^	United States	Fruit and vegetables; Convenience foods; Non-alcoholic beverages	5.2% (4)	No
Tru Blu Beverages Pty Ltd (Tru Blu Beverages)	Australia	Non-alcoholic beverages	2.3% (5)	No
Parmalat Australia Pty Ltd (Parmalat) ^6^	Italy	Dairy	2.2% (6)	No
**Total**			72.8%	
**Supermarkets**				
Woolworths Ltd (Au) (Woolworths)	Australia	Fruit and vegetables; Bread and bakery products; Convenience foods	34.2% (1)	Yes
Wesfarmers Ltd (Coles)	Australia	Bread and bakery products; Fruit and vegetables; Meat and meat products	29.8% (2)	Yes
IGA Inc (IGA)	Australia	Dairy; Confectionery; Bread and bakery products	7.0% (3)	Yes
Aldi Stores Supermarkets Pty Ltd (Aldi)	Germany	Dairy; Bread and bakery products; Fruit and vegetables	5.7% (4)	No
**Total**			76.7%	
**Quick service restaurants ^7^**				
McDonalds Corp (McDonalds)	United States	Burgers	27.9% (1)	No
Yum! Brands Inc (KFC and Pizza Hut)	United States	Chicken (*KFC*) Pizza (*Pizza Hut*)	12.5% (2)	No
Restaurant Brands International Inc (Hungry Jack’s)	Australia	Burgers	8% (3)	No
Doctor’s Associates Inc (Subway)	United States	Sandwiches	7.2% (4)	Yes
Domino’s Pizza (Domino’s Pizza)	United States	Pizza	4.6% (5)	No
Quick Service Restaurant Holdings Pty Ltd (Chicken Treat, Oporto, Red Rooster)	Australia	Chicken	4% (6)	No
Nando’s Group Holdings Ltd (Nando’s)	South Africa	Chicken	1.7% (7)	Yes
Grill’d (Grill’d)	Australia	Burgers	1.2% (8)	No
**Total**			67.1%	

^1^ For packaged food and beverage manufacturers and supermarkets, major product categories based on top three categories (based on number of products in portfolio) listed in “FoodSwitch: State of the Food Supply” 2019 report [33]. For supermarkets, product categories relate to supermarket “own-brand” product categories only. For quick service restaurants, major product categories based on a company’s “primary product portfolio” as defined in the “FoodSwitch: State of the Fast Food Supply” 2020 report [34]; ^2^ Sourced from Euromonitor Passport database for Australia, 2017 [29]; ^3^ Assessed using “supermarket” version of BIA-Obesity Australia 2018 tool; ^4^ Only one division of *Lion Pty Ltd*, *Lion Dairy and Drinks*, was assessed as part of this study; ^5^ *PepsiCo*’s snack brand is “*Smith’s Snackfood”*; ^6^ Included in the “packaged food manufacturer” sample; ^7^ Chains owned by the company and assessed for Australia listed in parenthesis.

**Table 2:** *BIA-Obesity Australia 2018*, domain-specific company scores by sector.

Company	Corporate Strategy	Product Formulation	Nutrition Labelling	Promotion Practices	Product Accessibility	Relationships with External Groups	Overall Score
	(%)	(%)	(%)	(%)	(%)	(%)	(out of 100)
**Packaged food and beverage manufacturers**							
Lion Dairy and Drinks	100	82	72	55	45	67	71
Nestlé	93	88	56	54	20	94	69
Unilever	93	68	75	56	50	88	68
Coca-Cola	90	86	52	43	20	88	64
Mars	78	68	75	52	15	80	64
Sanitarium	62	57	83	60	45	81	64
Simplot	63	59	71	57	40	94	62
Campbell Arnott’s	65	50	75	48	5	83	55
Fonterra	68	53	54	47	10	50	51
PepsiCo	72	53	19	46	0	25	50
Kellogg’s ^1^	63	50	64	41	0	38	48
George Weston Foods	63	41	47	39	0	75	44
Mondelēz ^1^	63	59	9	40	20	56	42
Kraft Heinz ^1^	55	38	54	0	0	25	29
McCain ^1^	0	12	19	20	0	19	14
Tru Blu Beverages ^1^	17	9	15	6	0	0	9
Schweppes ^1^	17	5	15	4	0	13	8
Goodman Fielder ^1^	0	0	18	0	0	0	4
Parmalat ^1^	0	0	6	0	0	44	3
Mean (SD)	56 (33)	46 (29)	46 (27)	35 (22)	14 (18)	54 (33)	43 (24)
Median	63	53	54	43	5	56	50
Q1	36	25	19	13	0	25	22
Q3	75	64	72	53	20	82	64
IQR	39	39	53	40	20	57	43
**Supermarkets**							
Woolworths	70	74	64	3	30	83	46
Coles	63	56	67	10	16	81	40
Aldi ^1^	55	15	3	0	2	13	11
IGA	0	18	10	0	0	44	8
Mean (SD)	47 (32)	41 (29)	36 (34)	3 (5)	12 (14)	55 (33)	26 (20)
Median	59	37	37	2	9	63	26
Q1	41	17	8	0	2	36	10
Q3	65	61	65	5	20	82	42
IQR	24	43	57	5	18	45	31
**Quick service restaurants**							
Subway	55	79	91	15	13	57	48
McDonald’s ^1^	52	33	82	49	11	44	42
KFC ^1^	48	44	82	41	9	13	41
Nando’s	17	11	55	44	13	NA ^2^	31
Hungry Jacks ^1^	33	22	82	24	0	13	28
Pizza Hut ^1^	33	8	82	36	0	9	27
Chicken Treat ^1^	0	0	27	35	5	0	14
Red Rooster ^1^	0	0	27	29	5	0	12
Oporto ^1^	0	0	27	25	5	0	11
Grill’d ^1^	17	17	18	0	8	6	10
Domino’s Pizza ^1^	0	0	14	0	0	13	3
Mean (SD)	23 (22)	19 (25)	53 (31)	27 (17)	6 (5)	16 (19)	24 (15)
Median	17	11	55	29	5	11	27
Q1	0	0	27	20	3	2	12
Q3	41	28	82	39	10	13	36
IQR	41	28	55	19	8	12	25

Table notes: ^1^ Assessment based on publicly available information only; ^2^ Nando’s explicitly stated that they had no relevant activity in this area and thus were not assessed in this domain; SD: standard deviation; IQR: interquartile range; NA: not applicable.

**Table 3:** Recommended actions for companies included in *BIA-Obesity Australia 2018*.

Domain	Recommended Action ^1^
Corporate strategy	Identify population nutrition and health as a priority focus area for the company, with relevant objectives, targets and appropriate resourcing (M, S, R) Refer to relevant international priorities (e.g., as articulated in the UN Sustainable Development Goals or the WHO Global NCD Action Plan) within the corporate strategy (M, S, R) Report progress against specific health and nutrition-related targets and objectives on a regular basis (M, S, R) Link the KPIs of senior management to nutrition and health-related targets in the corporate strategy (M, S, R)
Product formulation ^2^	Develop specific, time-bound category-specific targets for the reduction of nutrients of concern (sodium, sugar, saturated fat and artificially produced trans fat). Routinely report on progress in achieving reformulation targets (M, S, R) Limit or reduce energy content per serving / provide smaller package sizes in relevant product categories (e.g., ready meals, single-serve snacks) (M, S) Commit to reducing meal portion sizes by reducing kilojoule content of products and offering smaller/healthier sides and drinks as the default option (R) Participate in / implement a strategy to adopt relevant recommendations from government-led programs (e.g., Healthy Food Partnership) to improve the healthiness of the food supply (M, S, R) Actively work to increase servings of “five food groups” foods (e.g., vegetables, fresh fruit, wholegrains, reduced fat dairy) across key menu items (R) Commit to frying foods in non-hydrogenated, low saturated fat oils (R)
Nutrition labelling	Commit to full implementation of the Australian government-endorsed Health Star Rating system across all relevant products, with specific roll-out plan (M, S) ^2^ Introduce a policy to only make nutrition content claims (e.g., 99% fat free) on products classified as “healthy” (using government standards for classifying the healthiness of foods in relation to nutrition content claims) (M, S) ^2^ Provide comprehensive online nutrition information for all products (M, S, R) Provide comprehensive nutrition information at point of purchase (S and R) Commit to label artificially produced trans fat in relation to all relevant products (M, S) ^2^ Implement kilojoule labelling on menu boards across all states/territories and support the development of standardised interpretive nutrition labelling (e.g., using health stars or colour-coding) for menu boards (R) Clearly label healthier items on menu boards to make these options readily identifiable (R) Support the development of “free sugar” labelling regulations, e.g., through public in-principle support for “free sugar” labelling and commitment to implement ‘free sugar’ labelling once defined (M, S)
Promotion practices	Implement a policy for reducing the exposure of children and adolescents (up to the age of 18) to promotion of “less healthy” foods/brands that applies across all media channels, and includes all times/events when a large number of children/adolescents are likely to be exposed. Routinely report on compliance with the policy (M, S, R) Eliminate use of promotion techniques (e.g., cartoon characters, interactive games, toys in children’s meals) with strong appeal to children, including on product packaging (M, S, R) Commit to not sponsor sporting and community events that are popular with children/families using “unhealthy” products and brands (M, S, R) Increase the proportion of marketing activity that relates to healthier products and brands (if relevant) (M, S, R) Commit to increase the proportion of “healthy” products (using government guidelines* for classifying healthiness of foods) featured in catalogues and other advertising (S)
Product accessibility	Commit to increase the number and proportion of “healthy” products in the company’s portfolio (M, S) Commit to work with retailers to increase the prominence of healthier products relative to “less healthy” products in-store (e.g., through shelf space and strategic placement) and in promotional catalogues (M) Introduce universal healthy checkouts (with no “less healthy” products, such as confectionery and sugar-sweetened beverages, on display near registers) across all stores nationally (S) Limit price promotions (e.g., price discounts and “buy-one-get-one-free specials”) on “less healthy” products, whilst working to improve affordability of healthy foods (S) Increase the proportion of “healthy” products displayed in high-traffic areas (e.g., end-of-aisle displays) (S) Link rewards through loyalty programs to healthier purchases (S) Commit to make healthier and lower kilojoule meal options (e.g., healthier sides and drinks) the default option, particularly as part of children’s meals (R) Introduce pricing strategies that position healthier menu items at a similar or lower price to “less healthy” equivalents (where relevant), and restrict price promotions and value deal incentives on “less healthy” items (R) Promote healthier menu options (where relevant) through price discounts, promotions and/or loyalty bonuses on healthier items (R) Support the position of the WHO on fiscal policies to make healthier foods relatively cheaper and “less healthy” foods relatively more expensive, and make the company’s position public (M, S, R)
Relationships with external groups	Publish all relationships (including funding and support) with external groups (e.g., professional associations, research organisations, community and industry groups) related to health and nutrition (M, S, R) Disclose all political donations in real time, or commit to not make political donations (M, S, R)

^1^ The sector to which each recommended action applies is indicated by “M” for packaged food and beverage manufacturers, “S” for supermarkets, and “R” for quick service restaurants; ^2^ For supermarkets, relates to ‘own-brand’ products only.

## Supplementary Materials

The following are available online at https://www.mdpi.com/1660-4601/17/17/6118/s1, Table S1: BIA-Obesity Australia 2018 assessment tool for packaged food and beverage manufacturers; Table S2: BIA-Obesity Australia 2018 assessment tool for supermarkets; Table S3: BIA-Obesity Australia 2018 assessment tool for quick service restaurants; Table S4: Good practice examples by domain; File S1: Company scorecards (packaged food and beverage manufacturers, supermarkets, quick service restaurants).
