# Supplementary material for: Benchmarking the Nutrition-Related Policies and Commitments of Major Food Companies in Australia, 2018
Source: Int J Environ Res Public Health. 2020 Aug 22;17(17):6118. doi: 10.3390/ijerph17176118 (PMC7504100; doi:10.3390/ijerph17176118)
Supplement: Supplementary file 1 [file ijerph-17-06118-s001.zip › File S1 - Company scorecards IJERPH.pdf]

International Journal of
Environmental Research
and Public Health

Supplementary material

File S1: Company scorecards

- Packaged food and beverage manufacturers (pages 2-20)
- Supermarkets (pages 21-24)
- Quick Service Restaurants (25-35)

Campbell Arnott's

8th OUT OF 19 FOOD AND BEVERAGE MANUFACTURERS

55 OVERALL SCORE (OUT OF 100)

Domain	Weighting
A Corporate strategy	10%
B Product formulation	30%
C Nutrition labelling	20%
D Promotion practices	30%
E Product accessibility	5%
F Relationships with external groups	5%

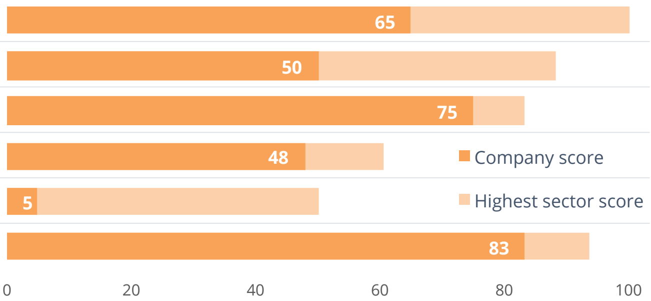

Areas of strength

A| Corporate strategy - Campbell Arnott's identifies nutrition as an area of focus for the company, and reports against some of its progress in achieving nutrition targets on its website and through global corporate social responsibility reports

B| Product formulation – Campbell Arnott's has set internal reformulation targets across portions of its portfolio, and is a participant in the Australian government's Healthy Food Partnership

C| Nutrition labelling - Campbell Arnott's commits to full implementation of the Australian government-endorsed Health Star Rating system across the company's portfolio, and provides comprehensive online nutrition information for its products, both per serve and per 100g

F| Relationships with external groups - Campbell Arnott's publishes its support for professional associations related to nutrition and health

Prioritised recommended actions for Campbell Arnott's

1| **Elevate** nutrition and health to a higher priority focus area within the business, with appropriate resourcing, objectives and targets

2| **Implement** a strengthened policy for reducing promotion of 'less healthy' products/brands that applies to children and adolescents (**up to the age of 18**), across all media channels, and includes all times/events when a large number of children/adolescents are likely to be exposed. Adopt government guidelines for classifying the healthiness of foods

3| **Develop** and publicise specific, time-bound targets for the reduction of sodium, free sugar, saturated fat, and artificially produced trans fat across the product portfolio. Routinely report on progress in achieving reformulation targets

4| **Eliminate** use of promotion techniques (e.g., cartoon characters, interactive games) with strong appeal to children in relation to 'less healthy' products and brands

5| **Publicly** commit to only make nutrition content claims (e.g., "99% fat free") on products that are classified as 'healthy' (using government standards for classifying the healthiness of foods in relation to health claims)

6| **Commit** to work with retailers to increase the prominence of healthier products relative to 'less healthy' products in-store (e.g., through shelf space and strategic placement) and in promotional catalogues

Coca-Cola

4th OUT OF 19 FOOD AND BEVERAGE MANUFACTURERS

64 OVERALL SCORE (OUT OF 100)

Domain	Weighting
A Corporate strategy	10%
B Product formulation	30%
C Nutrition labelling	20%
D Promotion practices	30%
E Product accessibility	5%
F Relationships with external groups	5%

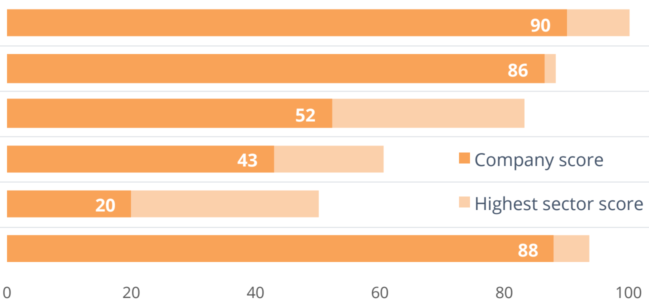

Areas of strength

A| Corporate strategy - Coca-Cola identifies nutrition and health as a focus area for the company, and reports annually against progress in achieving its nutrition and health objectives at the national level

B| Product formulation - Coca-Cola has set specific, time-bound targets for the reduction of added sugars and kilojoule content across the company's portfolio, and commits to offer smaller pack sizes across the portfolio

E| Product accessibility - Coca-Cola Australia has set a clear target that all new product innovations will be low or no sugar options

F| Relationships with external groups - Coca-Cola discloses a list of the external groups, organisations and research it supports at the national and global level, including funding amounts. Details are available in a consolidated and accessible format, and updated annually

Prioritised recommended actions for Coca-Cola

1| **Implement** a strengthened policy for reducing promotion of 'less healthy' products/brands that applies to children and adolescents **(up to the age of 18)**, across all media channels, and includes all times/events when a large number of children/adolescents are likely to be exposed. Adopt government guidelines for classifying the healthiness of foods

2| **Commit** not to sponsor sporting and community events that are popular with children/families using 'less healthy' products and brands

3| **Eliminate** use of promotion techniques (e.g., cartoon characters, interactive games) with strong appeal to children in relation to 'less healthy' products and brands

4| **Commit** to implement interpretive HSR labelling (use of Health Stars, rather than just the energy icon) across all relevant products

5| **Commit** to work with retailers to increase the prominence of healthier products relative to 'less healthy' products in-store (e.g., through shelf space and strategic placement) and in promotional catalogues

6| **Commit** to not directly supply any school in Australia with full sugar carbonated beverages

Fonterra

9th OUT OF 19 FOOD AND BEVERAGE MANUFACTURERS

51 OVERALL SCORE (OUT OF 100)

Domain	Weighting
**A** Corporate strategy	10%
**B** Product formulation	30%
**C** Nutrition labelling	20%
**D** Promotion practices	30%
**E** Product accessibility	5%
**F** Relationships with external groups	5%

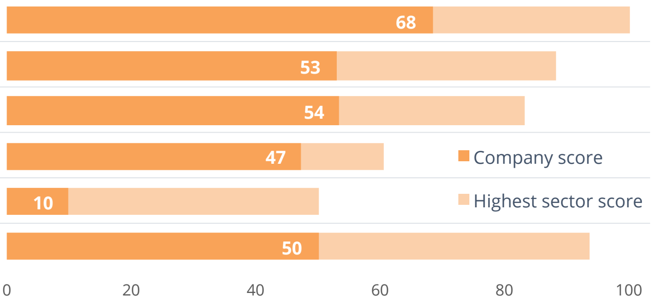

Areas of strength

A| **Corporate strategy** - Fonterra highlights nutrition as an area of focus for the company through global sustainability reporting. The company references priorities laid out by the WHO and the UN Sustainable Development Goals

B| **Product formulation** – Fonterra commits to reduce levels of added sugars, sodium and total fat across portions of its global portfolio

C| **Nutrition labelling** - Fonterra provides comprehensive online nutrition information for its products, both per serve and per 100g

Prioritised recommended actions for Fonterra

1| **Establish** national reporting (e.g., in annual review, on website) of progress against specific Australian nutrition and health objectives and targets

2| **Develop** and publicise specific, time-bound targets for the reduction of sodium, free sugar, saturated fat and kilojoule content, across the Australian product portfolio. Routinely report on and externally audit progress in achieving reformulation targets

3| **Publicly commit** to implement the Health Star Rating system across all products in the Australian product portfolio, with specific roll-out plan

4| **Implement** a strengthened policy for reducing promotion of 'less healthy' products/brands that applies to children and adolescents (**up to the age of 18**), across all media channels, and includes all times/events when a large number of children/adolescents are likely to be exposed. Adopt government guidelines for classifying the healthiness of foods

5| **Commit** to work with retailers to increase the prominence of healthier products relative to 'less healthy' products in-store (e.g., through shelf space and strategic placement) and in promotional catalogues

George Weston Foods

12th OUT OF 19 FOOD AND BEVERAGE MANUFACTURERS

44 OVERALL SCORE (OUT OF 100)

Domain	Weighting
**A** Corporate strategy	10%
**B** Product formulation	30%
**C** Nutrition labelling	20%
**D** Promotion practices	30%
**E** Product accessibility	5%
**F** Relationships with external groups	5%

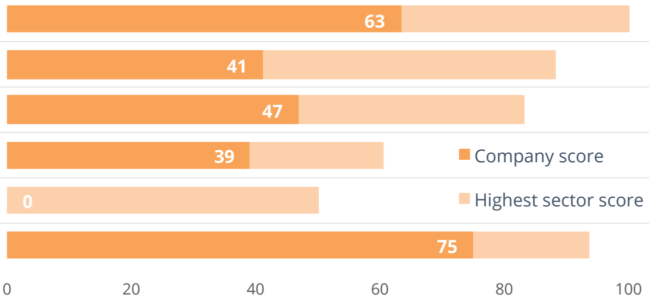

Areas of strength

A| **Corporate strategy** - George Weston Foods makes some commitment to nutrition through national and global corporate reporting

B| **Product formulation** - George Weston Foods commits to reduce sodium across its product portfolio

C| **Nutrition labelling** - George Weston Foods commits to implementing the Australian government-endorsed Health Star Rating system across a portion of its portfolio, and provides comprehensive online nutrition information for its products

Prioritised recommended actions for George Weston Foods

1| **Identify** nutrition and health as a priority focus area for the company, with relevant objectives, targets and appropriate resourcing. Report progress against specific nutrition and health targets and objectives at the national level

2| **Implement** a strengthened policy for reducing promotion of 'less healthy' products/brands that applies to children and adolescents (**up to the age of 18**), across all media channels, and includes all times/events when a large number of children /adolescents are likely to be exposed. Adopt government guidelines for classifying the healthiness of foods

3| **Develop** specific, time-bound targets for the reduction of sodium, free sugar and saturated fat across the product portfolio. Routinely report on progress in achieving reformulation targets

4| **Participate** in / implement a strategy to adopt relevant recommendations from government-led programs (e.g., Healthy Food Partnership) to improve the healthiness of the food supply

5| **Commit** to implement the Health Star Rating system across products from all divisions of the business, with specific roll-out plan

Goodman Fielder*

18th OUT OF 19 FOOD AND BEVERAGE MANUFACTURERS

4 OVERALL SCORE (OUT OF 100)

Domain	Weighting
**A** Corporate strategy	10%
**B** Product formulation	30%
**C** Nutrition labelling	20%
**D** Promotion practices	30%
**E** Product accessibility	5%
**F** Relationships with external groups	5%

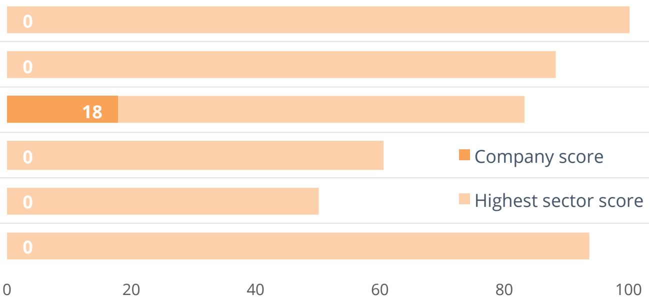

Areas of strength

C) Nutrition labelling - Goodman Fielder provides comprehensive nutrition information online for the majority of its products, both per serve and per 100g

Goodman Fielder had little disclosure of its approach to nutrition and health. As such no further areas of strength were identified.

*Assessment based on publicly available information only

Prioritised recommended actions for Goodman Fielder

1| **Identify** nutrition and health as a priority focus area for the company, with relevant objectives, targets and appropriate resourcing

2| **Develop** specific, time-bound targets for the reduction of nutrients of concern (sodium, free sugar, saturated fat, and artificially produced trans fat) across the product portfolio. Routinely report on progress in achieving reformulation targets

3| **Participate** in / implement a strategy to adopt relevant recommendations from government-led programs (e.g., Healthy Food Partnership) to improve the healthiness of the food supply

4| **Commit** to full implementation of the Australian government-endorsed Health Star Rating system across all relevant products, with specific roll-out plan

5| **Implement** a policy for reducing promotion of 'less healthy' products/brands that applies to children and adolescents (**up to the age of 18**), across all media channels, and includes all times/events when a large number of children/adolescents are likely to be exposed. Adopt government guidelines for classifying the healthiness of foods

6| **Commit** to work with retailers to increase the prominence of healthier products relative to 'less healthy' products in-store (e.g., through shelf space and strategic placement) and in promotional catalogues

Kellogg's*

11th OUT OF 19 FOOD AND BEVERAGE MANUFACTURERS

48 OVERALL SCORE (OUT OF 100)

Domain	Weighting
**A** Corporate strategy	10%
**B** Product formulation	30%
**C** Nutrition labelling	20%
**D** Promotion practices	30%
**E** Product accessibility	5%
**F** Relationships with external groups	5%

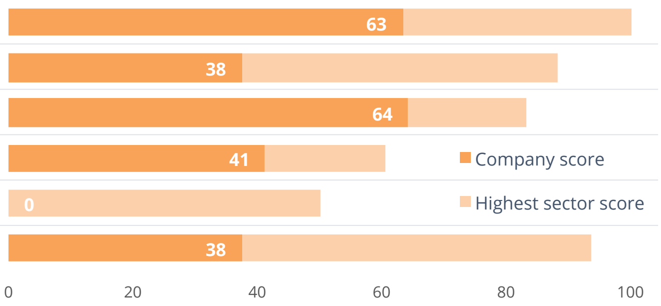

Areas of strength

A| **Corporate strategy** - At the global level, Kellogg's identifies nutrition and health as an area of focus for the company and references priorities laid out by the UN Sustainable Development Goals

B| **Product formulation** - Kellogg's Australia commits to providing healthier options through reformulation of its portfolio. Globally, the company reports on some of its progress in reformulating products to reduce levels of sodium, artificially produced trans fat and sugars

C| **Nutrition labelling** - Kellogg's Australia commits to implement the government-endorsed Health Star Rating system across all products in the company's portfolio, with a specific roll out plan. Kellogg's Australia provides comprehensive nutrition information online for its products

*Assessment based on publicly available information only

Prioritised recommended actions for Kellogg's

1| **Identify** nutrition and health as a priority focus area for the company at the national level, and develop specific objectives that are reported against at the national level

2| **Implement** a strengthened policy for reducing promotion of 'less healthy' products/brands that applies to children and adolescents (**up to the age of 18**), across all media channels, and includes all times/events when a large number of children/adolescents are likely to be exposed. Adopt government guidelines for classifying the healthiness of foods

3| Publicly **commit** to Australia-specific, time-bound targets for the reduction of sodium, free sugar, saturated fat and artificially produced trans fat as well as portion size/energy content across the product portfolio. Routinely report on progress in achieving reformulation targets

4| **Commit** not to sponsor sporting and community events that are popular with children/families using 'less healthy' products and brands

5| **Extend** commitment to eliminate use of promotion techniques with strong appeal to children in relation to 'less healthy' products and brands, by ensuring commitment applies also to product packaging

6| **Publicly** commit to only make nutrition content claims (e.g., "99% fat free") on products that are classified as 'healthy' (using government standards for classifying the healthiness of foods in relation to health claims

7| **Commit** to work with retailers to increase the prominence of healthier products relative to 'less healthy' products in-store (e.g., through shelf space and strategic placement) and in promotional catalogues

Kraft Heinz*

14th OUT OF 19 FOOD AND BEVERAGE MANUFACTURERS

29 OVERALL SCORE (OUT OF 100)

Domain	Weighting
**A** Corporate strategy	10%
**B** Product formulation	30%
**C** Nutrition labelling	20%
**D** Promotion practices	30%
**E** Product accessibility	5%
**F** Relationships with external groups	5%

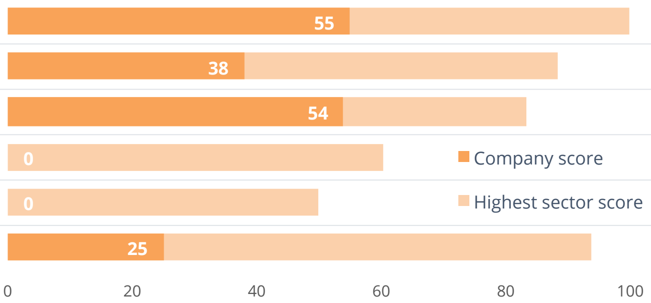

Areas of strength

A| **Corporate strategy** - Kraft Heinz identifies nutrition and health as an area of focus for the company at the national level, and reports against some of its progress in achieving its nutrition and health objectives

B| **Product formulation** – Kraft Heinz reports against progress in achieving sodium and sugar reductions across its portfolio

C| **Nutrition labelling** – Kraft Heinz commits to implement the Australian government-endorsed Health Star Rating system across a number of products in its portfolio, and provides comprehensive online nutrition information for products

*Assessment based on publicly available information only

Prioritised recommended actions for Kraft Heinz

1| **Elevate** nutrition and health to a priority focus within the business, with appropriate resourcing, and relevant objectives and targets

2| **Implement** a policy for reducing promotion of 'less healthy' products/brands that applies to children and adolescents (**up to the age of 18)**, across all media channels, and includes all times/events when a large number of children/adolescents are likely to be exposed. Adopt government guidelines for classifying the healthiness of foods

3| **Develop** and publicise specific, time-bound targets for the reduction of saturated fat, artificially produced trans fat and kilojoule content across the product portfolio. Develop targets for the reduction of sodium and free sugars for products that do not already meet the company's healthy standard. Routinely report on progress in achieving reformulation targets

4| **Participate** in / implement a strategy to adopt relevant recommendations from government-led programs (e.g., Healthy Food Partnership) to improve the healthiness of the food supply

5| **Commit** to work with retailers to increase the prominence of healthier products relative to 'less healthy' products in-store (e.g., through shelf space and strategic placement) and in promotional catalogues

6| **Publicly** commit to only make nutrition content claims (e.g., "99% fat free") on products that are classified as 'healthy' (using government standards for classifying the healthiness of foods in relation to health claims)

Lion Dairy & Drinks

1st OUT OF 19 FOOD AND BEVERAGE MANUFACTURERS

71 OVERALL SCORE (OUT OF 100)

Domain	Weighting
**A** Corporate strategy	10%
**B** Product formulation	30%
**C** Nutrition labelling	20%
**D** Promotion practices	30%
**E** Product accessibility	5%
**F** Relationships with external groups	5%

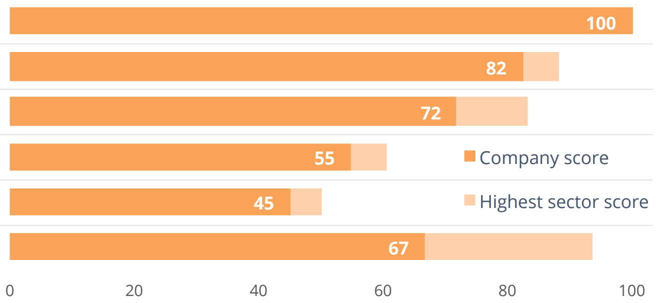

Areas of strength

A| **Corporate strategy** - Lion Dairy & Drinks highlights nutrition and health as a key focus area for the company, and aligns their approach with Australian government guidelines and priorities set out by the WHO. The company reports annually against nutrition and health objectives at the national level

B| **Product formulation** - Lion Dairy & Drinks has set clear, time-bound reformulation targets for the reduction of sodium, added sugars, fat and kilojoule content across the majority of its portfolio, and has set specific reformulation targets for children's products. The company commits not to use any artificially produced trans fats in its products. Lion Dairy & Drinks is a participant in the Australian government's Healthy Food Partnership

C| **Nutrition labelling** - Lion Dairy & Drinks commits to implement the Australian government-endorsed HSR system across all products in the company's portfolio, and provides comprehensive online nutrition information for the majority of its products

E| **Product accessibility** - Lion Dairy & Drinks commits to transition a portion of its portfolio into a 'healthier' product category, and to work with retailers to position healthier products at the front of the store

Prioritised recommended actions for Lion Dairy & Drinks

1| Implement a strengthened policy for reducing promotion of 'less healthy' products/brands that applies to children and adolescents **(up to the age of 18)**, across all media channels, and includes all times/events when a large number of children/adolescents are likely to be exposed. Continue using government guidelines for classifying the healthiness of foods, and routinely report on compliance with the policy

2| **Commit** not to sponsor sporting and community events that are popular with children/families using 'less healthy' products and brands

3| **Limit** or reduce energy content per serving in relevant product categories (e.g., single-serve snacks/drinks)

4| **Publicly** commit to only make nutrient content claims (e.g., "99% fat free") on products that are classified as 'healthy' (using government standards for classifying the healthiness of foods in relation to health claims)

5| **Strengthen** the company's commitment to work with retailers to increase the prominence of healthier products relative to 'less healthy' products in-store, by expanding scope to include product placement throughout the store, and promotion in catalogues

6| **Support** the development of 'free sugar' labelling regulations

Mars

4th OUT OF 19 FOOD AND BEVERAGE MANUFACTURERS

64 OVERALL SCORE (OUT OF 100)

Domain	Weighting
**A** Corporate strategy	10%
**B** Product formulation	30%
**C** Nutrition labelling	20%
**D** Promotion practices	30%
**E** Product accessibility	5%
**F** Relationships with external groups	5%

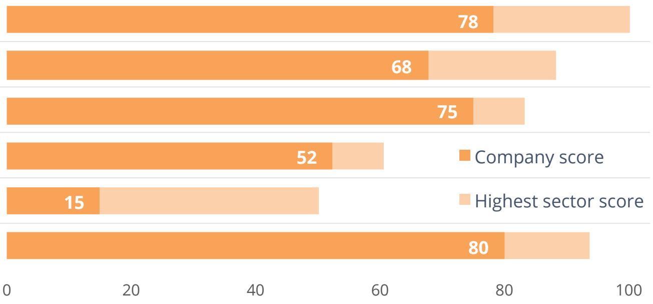

Areas of strength

A| Corporate strategy - Mars highlights nutrition and health as a priority focus area in company reporting. At the global level, the company provides detail on its progress in achieving nutrition and health objectives, and references priorities laid out by the WHO

B| Product formulation - Mars has set specific, time-bound targets for the reduction of saturated fat, sodium and kilojoule content across portions of its portfolio. Across its confectionery business unit, the company commits to increase the number of single serve products and move away from 'king size'

C| Nutrition labelling - Mars commits to implement the Australian government-endorsed Health Star Rating system across its food and confectionery range

E| Product accessibility - Mars makes a clear commitment to increase the number of 'healthy' products in the food segment of its portfolio

Prioritised recommended actions for Mars

1| **Introduce** national-level reporting against specific nutrition and health targets and objectives

2| **Implement** a strengthened policy for reducing promotion of 'less healthy' products/brands that applies to children and adolescents (**up to the age of 18**), across all media channels, and includes all times/events when a large number of children/adolescents are likely to be exposed. Adopt government guidelines for classifying the healthiness of foods

3| **Develop** specific, time-bound targets for the reduction of saturated fat and free sugars across all relevant segments of the product portfolio

4| **Commit** not to sponsor sporting and community events that are popular with children/families using 'less healthy' products and brands

5| **Extend** commitment to eliminate use of promotion techniques with strong appeal to children in relation to 'less healthy' products and brands, by ensuring commitment applies also to product packaging

6| **Commit** to work with retailers to increase the prominence of healthier products relative to 'less healthy' products in-store (e.g., through shelf space and strategic placement) and in promotional catalogues

COMPANY SCORECARDS
PACKAGED FOOD AND BEVERAGE MANUFACTURERS

McCain*

15th OUT OF 19 FOOD AND BEVERAGE MANUFACTURERS

14 OVERALL SCORE (OUT OF 100)

Domain	Weighting
**A** Corporate strategy	10%
**B** Product formulation	30%
**C** Nutrition labelling	20%
**D** Promotion practices	30%
**E** Product accessibility	5%
**F** Relationships with external groups	5%

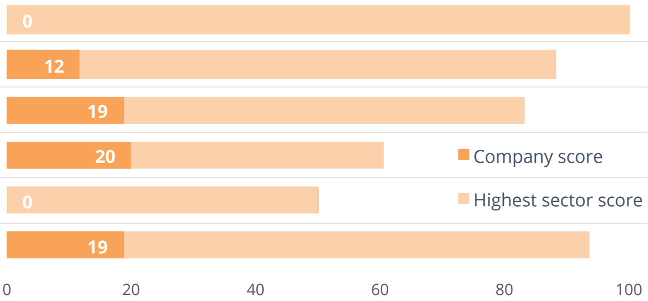

Areas of strength

B| Product formulation – McCain makes a general commitment at the global level to reduce use of sugar, salt and fat in its products

C| Nutrition labelling - McCain provides comprehensive nutrition information online for its products, both per serve and per 100g

McCain had little disclosure of its approach to nutrition and health at the national level. As such no further areas of strength were identified.

*Assessment based on publicly available information only

Prioritised recommended actions for McCain

1| **Identify** nutrition and health as a priority focus area for the company, with relevant objectives, targets and appropriate resourcing. Report progress against specific nutrition and health objectives at the national level

2| **Implement** a policy for reducing promotion of 'less healthy' products/brands that applies to children and adolescents (**up to the age of 18**), across all media channels, and includes all times/events when a large number of children /adolescents are likely to be exposed. Adopt government guidelines for classifying the healthiness of foods

3| **Develop** specific, time-bound targets for the reduction of sodium, free sugar, saturated fat, artificially produced trans fat and kilojoule content across the portfolio. Routinely report on progress in achieving reformulation targets

4| **Participate** in / implement a strategy to adopt relevant recommendations from government-led programs (e.g., Healthy Food Partnership) to improve the healthiness of the food supply

5| **Commit** to full implementation of the Australian government-endorsed Health Star Rating system across all relevant products, with specific roll-out plan

6| **Commit** to work with retailers to increase the prominence of healthier products relative to 'less healthy' products in-store (e.g., through shelf space and strategic placement) and in promotional catalogues

COMPANY SCORECARDS
PACKAGED FOOD AND BEVERAGE MANUFACTURERS

Mondelēz*

13th OUT OF 19 FOOD AND BEVERAGE MANUFACTURERS

42 OVERALL SCORE (OUT OF 100)

Domain	Weighting
**A** Corporate strategy	10%
**B** Product formulation	30%
**C** Nutrition labelling	20%
**D** Promotion practices	30%
**E** Product accessibility	5%
**F** Relationships with external groups	5%

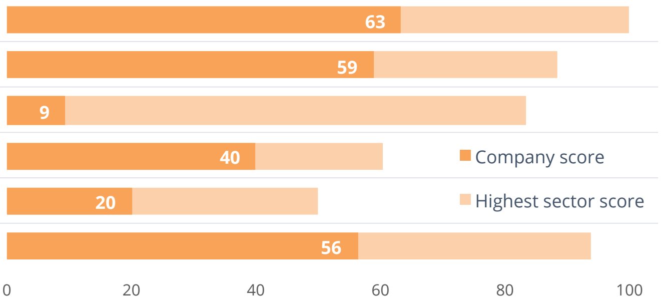

Areas of strength

A| **Corporate strategy** - Mondelēz identifies nutrition and health as an area of focus for the company at the global level. Globally, it reports against some of its nutrition and health objectives and references priorities laid out by the WHO

B| **Product formulation** - Mondelēz has set global reformulation targets to reduce levels of sodium, artificially produced trans fat and saturated across the company's portfolio

E| **Product accessibility** - Mondelēz makes a commitment that 25% of its revenue will come from its 'healthier' product range by 2020

*Assessment based on publicly available information only

Prioritised recommended actions for Mondelēz

1| **Elevate** nutrition and health as a priority focus area within the business at the national level, and report on progress against Australia-specific health and nutrition objectives

2| **Implement** a strengthened policy for reducing promotion of 'less healthy' products/brands that applies to children and adolescents (**up to the age of 18**), across all media channels, and includes all times/events when a large number of children /adolescents are likely to be exposed. Adopt government guidelines for classifying the healthiness of foods

3| **Develop** and publicise specific, time-bound reformulation targets for the reduction of free sugars across the company's portfolio

4| **Commit** to full implementation of the Health Star Rating system across all relevant products, with specific roll-out plan

5| **Provide** comprehensive online information for all products in the company's portfolio

6| **Commit** not to sponsor sporting and community events that are popular with children/families using 'less healthy' products and brands

7| **Eliminate** use of promotion techniques (e.g., cartoon characters, interactive games) with strong appeal to children in relation to 'less healthy' products and brands

8| **Commit** to work with retailers to increase the prominence of healthier products relative to 'less healthy' products in-store (e.g., through shelf space and strategic placement) and in promotional catalogues

Nestlé

2nd OUT OF 19 FOOD AND BEVERAGE MANUFACTURERS

69 OVERALL SCORE (OUT OF 100)

Domain	Weighting
**A** Corporate strategy	10%
**B** Product formulation	30%
**C** Nutrition labelling	20%
**D** Promotion practices	30%
**E** Product accessibility	5%
**F** Relationships with external groups	5%

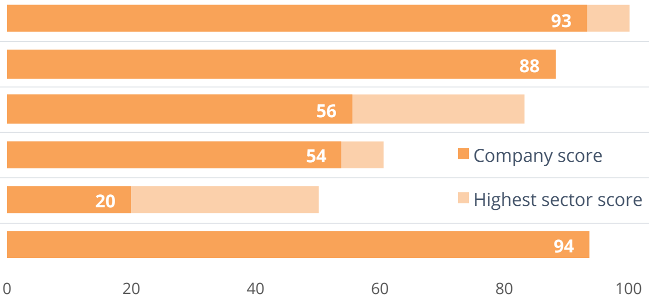

Areas of strength

A| **Corporate strategy** - Nestlé identifies nutrition and health as a priority focus area for the company, setting clear nutrition objectives and targets. The company reports against its nutrition and health commitments annually at both the national and global level

B| **Product formulation** - Nestlé has developed specific, time-bound targets for the reduction of sodium, added sugar and saturated fat across all products in its portfolio, and has reported the complete removal of artificially produced trans fats. The company operates transparently in its approach to reformulation, tracking and disclosing progress in achieving targets on an annual basis. Nestlé is a participant in the Australian government's Healthy Food Partnership

C| **Nutrition labelling** - Nestlé commits to implement the Australian government-endorsed HSR system across the company's portfolio, and provides comprehensive online nutrition information for some of its products

F| **Relationships with external groups** - Nestlé adopts good disclosure of its support for external groups related to nutrition and health, and implements a global policy that prohibits political donations

Prioritised recommended actions for Nestlé

1| **Implement** a strengthened policy for reducing promotion of 'less healthy' products/brands that applies to children and adolescents (**up to the age of 18**), across all media channels, and includes all times/events when a large number of children/adolescents are likely to be exposed. Adopt government guidelines for classifying the healthiness of foods

2| **Commit** not to sponsor sporting and community events that are popular with children/families using 'less healthy' products and brands

3| **Extend** commitment to eliminate use of promotion techniques with strong appeal to children in relation to 'less healthy' products and brands, by ensuring commitment applies also to product packaging

4| **Publicly** commit to only make nutrition content claims (e.g., "99% fat free") on products that are classified as 'healthy' (using government standards for classifying the healthiness of foods in relation to health claims)

5| **Commit** to work with retailers to increase the prominence of healthier products relative to 'less healthy' products in-store (e.g., through shelf space and strategic placement) and in promotional catalogues

6| **Support** the development of 'free sugar' labelling regulations

Parmalat*

19th OUT OF 19 FOOD AND BEVERAGE MANUFACTURERS

3 OVERALL SCORE (OUT OF 100)

Domain	Weighting
**A** Corporate strategy	10%
**B** Product formulation	30%
**C** Nutrition labelling	20%
**D** Promotion practices	30%
**E** Product accessibility	5%
**F** Relationships with external groups	5%

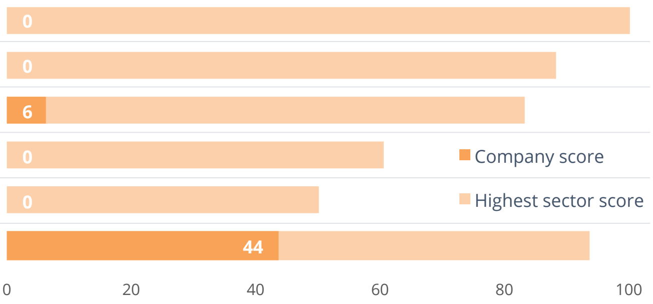

Areas of strength

Parmalat had little disclosure of its approach to nutrition and health. As such, no clear areas of strength were identified.

Prioritised recommended actions for Parmalat

1| **Identify** nutrition and health as a priority focus area for the company, with relevant objectives, targets and appropriate resourcing

2| **Develop** specific, time-bound targets for the reduction of nutrients of concern (sodium, free sugar, saturated fat, and artificially produced trans fat) across the product portfolio. Routinely report on progress in achieving reformulation targets

3| **Participate** in / implement a strategy to adopt relevant recommendations from government-led programs (e.g., Healthy Food Partnership) to improve the healthiness of the food supply

4| **Commit** to full implementation of the Australian government-endorsed Health Star Rating system across all relevant products, with specific roll-out plan

5| **Implement** a policy for reducing promotion of 'less healthy' products/brands that applies to children and adolescents (**up to the age of 18**), across all media channels, and includes all times/events when a large number of children/adolescents are likely to be exposed. Adopt government guidelines for classifying the healthiness of foods

6| **Commit** to work with retailers to increase the prominence of healthier products relative to 'less healthy' products in-store (e.g., through shelf space and strategic placement) and in promotional catalogues

*Assessment based on publicly available information only

PepsiCo

10th OUT OF 19 FOOD AND BEVERAGE MANUFACTURERS

50 OVERALL SCORE (OUT OF 100)

Domain	Weighting
**A** Corporate strategy	10%
**B** Product formulation	30%
**C** Nutrition labelling	20%
**D** Promotion practices	30%
**E** Product accessibility	5%
**F** Relationships with external groups	5%

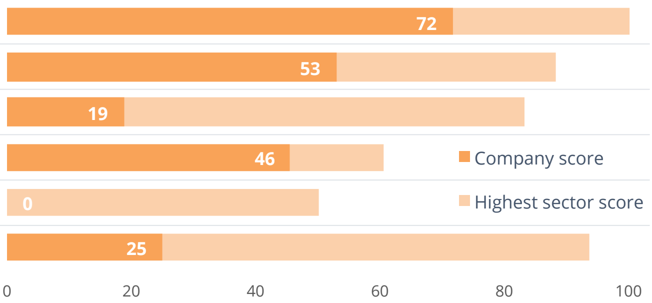

Areas of strength

A| **Corporate strategy** - PepsiCo Australia makes a commitment to nutrition and health through the company's website and in global corporate reporting

B| **Product formulation** - PepsiCo Australia reports reductions in levels of saturated fat, artificially produced trans fat and sodium across its snack portfolio. At the global level, PepsiCo has set specific, time-bound targets to reduce sodium, artificially produced trans fat, saturated fat and added sugars across portions of its portfolio

C| **Nutrition labelling** - PepsiCo Australia provides comprehensive online nutrition information for all of its products (per serve and per 100g) and includes Health Star Ratings for products online

Prioritised recommended actions for PepsiCo

1| **Implement** a strengthened policy for reducing promotion of 'less healthy' products/brands that applies to children and adolescents (**up to the age of 18**), across all media channels, and includes all times/events when a large number of children/adolescents are likely to be exposed. Adopt government guidelines for classifying the healthiness of foods

2| **Commit** not to sponsor sporting and community events that are popular with children/families using 'less healthy' products and brands

3| **Publicly commit** to full implementation of the Health Star Rating system across all relevant products, with specific roll-out plan. As part of this commitment, implement interpretive HSR labelling (use of Health Stars, rather than just the energy icon)

4| **Develop** specific, time-bound targets for the reduction of sodium, free sugar, saturated fat and kilojoule content across the Australian snacks and beverages portfolio (where relevant to product category). Routinely report on progress in achieving reformulation targets

5| **Commit** to work with retailers to increase the prominence of healthier products relative to 'less healthy' products in-store (e.g., through shelf space and strategic placement) and in promotional catalogues

6| **Commit** to not directly supply any school in Australia with full sugar carbonated beverages or energy drinks

Sanitarium

4th OUT OF 19 FOOD AND BEVERAGE MANUFACTURERS

64 OVERALL SCORE (OUT OF 100)

Domain	Weighting
**A** Corporate strategy	10%
**B** Product formulation	30%
**C** Nutrition labelling	20%
**D** Promotion practices	30%
**E** Product accessibility	5%
**F** Relationships with external groups	5%

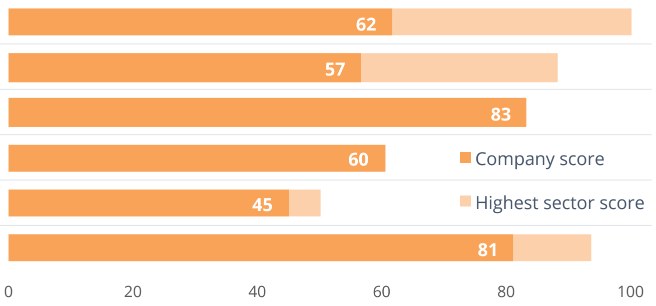

Areas of strength

A| Corporate strategy - Sanitarium clearly communicates its commitment to nutrition and health though the company's overarching mission statement, and highlights nutrition and health-related issues as a key priority within the business

C| Nutrition labelling - Sanitarium outlines its support for the Australian government-endorsed Health Star Rating system, and commits to full implementation of HSR labelling across all products in its portfolio. Sanitarium provides comprehensive online nutrition information for all its products, including per serve, per 100g and HSR

D| Promotion practices
Sanitarium identifies responsible marketing to children as a company priority and commits to only market 'healthy' foods to children

E| Product accessibility – Sanitarium has a broad commitment to encourage healthy eating and provide healthy and nutritious products to all consumers

Prioritised recommended actions for Sanitarium

1| Report progress against specific nutrition and health objectives at the national level on a regular basis

2| Implement a strengthened policy for reducing promotion of 'less healthy' products/brands that applies to children and adolescents (**up to the age of 18**), across all media channels, and includes all times/events when a large number of children/adolescents are likely to be exposed. Continue using government guidelines for classifying the healthiness of foods

3| Develop and publicise specific, time-bound targets for the reduction of sodium, free sugar and saturated fat across the product portfolio, and commit to reducing the kilojoule content of single-serve products. Routinely report on progress in achieving reformulation targets

4| Commit to work with retailers to increase the prominence of healthier products relative to 'less healthy' products in-store (e.g., through shelf space and strategic placement) and in promotional catalogues

5| Support the development of 'free sugar' labelling regulations

Schweppes*

17th OUT OF 19 FOOD AND BEVERAGE MANUFACTURERS

8 OVERALL SCORE (OUT OF 100)

Domain	Weighting
**A** Corporate strategy	10%
**B** Product formulation	30%
**C** Nutrition labelling	20%
**D** Promotion practices	30%
**E** Product accessibility	5%
**F** Relationships with external groups	5%

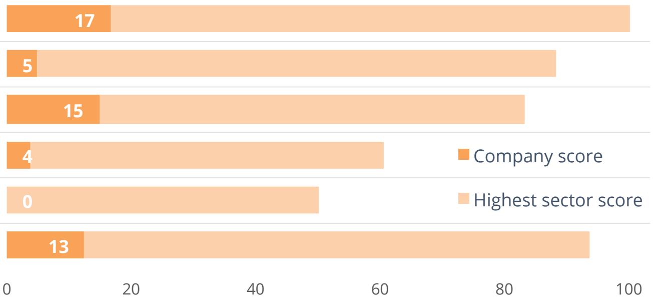

Areas of strength

B| Product formulation - Schweppes makes some commitment to reduce sugar in its products at the global level

C| Nutrition labelling - Schweppes provides comprehensive nutrition information online for its products, both per serve and per 100g

Schweppes had little disclosure of its approach to nutrition and health. As such no further areas of strength were identified.

*Assessment based on publicly available information only

Prioritised recommended actions for Schweppes

1| **Identify** nutrition and health as a priority focus area for the company, with relevant objectives, targets and appropriate resourcing. Report progress against specific nutrition and health objectives

2| **Develop** specific, time-bound targets for the reduction of free sugar and kilojoule content across the product portfolio. Routinely report on progress in achieving reformulation targets

3| **Publicly commit** to full implementation of the Health Star Rating system across all relevant products, with specific roll-out plan. As part of this commitment, implement interpretive HSR labelling (use of Health Stars, rather than just the energy icon)

4| **Develop** a strategy to increase the proportion of 'healthy' products in the company's portfolio

5| **Commit** to not directly supply any school in Australia with full sugar carbonated beverages or energy drinks

6| **Implement** a policy for reducing promotion of 'less healthy' products/brands that applies to children and adolescents (**up to the age of 18**), across all media channels, and includes all times/events when a large number of children /adolescents are likely to be exposed. Adopt government guidelines for classifying the healthiness of foods

7| **Commit** to work with retailers to increase the prominence of healthier products relative to 'less healthy' products in-store (e.g., through shelf space and strategic placement) and in promotional catalogues

Simplot

7th OUT OF 19 FOOD AND BEVERAGE MANUFACTURERS

62 OVERALL SCORE (OUT OF 100)

Domain	Weighting
**A** Corporate strategy	10%
**B** Product formulation	30%
**C** Nutrition labelling	20%
**D** Promotion practices	30%
**E** Product accessibility	5%
**F** Relationships with external groups	5%

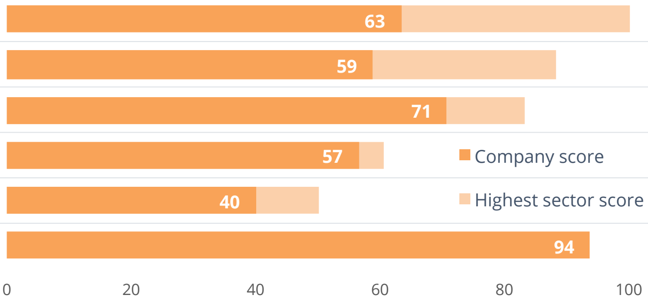

Areas of strength

A| **Corporate strategy** - Simplot identifies nutrition and health as an area of focus for the company, and reports against some of its progress in achieving nutrition and health objectives

B| **Product formulation** - Simplot has set internal reformulation targets for the reduction of nutrients of concern across the portfolio

C| **Nutrition labelling** - Simplot commits to full implementation of the Australian government-endorsed Health Star Rating system across all products in its portfolio, and reports on implementation of HSR labelling on its website

E| **Product accessibility** - Simplot has set a clear, time-bound target to transition a portion of its portfolio into a 'healthier' category

Prioritised recommended actions for Simplot

1| **Communicate** Simplot's approach to nutrition and health-related issues in corporate reporting, and report progress against specific nutrition and health objectives on a regular basis

2| **Implement** a strengthened policy for reducing promotion of 'less healthy' products/brands that applies to children and adolescents (**up to the age of 18**), across all media channels, and includes all times/events when a large number of children/adolescents are likely to be exposed. Continue using government guidelines for classifying the healthiness of foods

3| **Publicise** specific, time-bound targets for the reduction of nutrients of concern across the portfolio, and routinely report against progress in achieving reformulation targets

4| **Commit** to work with retailers to increase the prominence of healthier products relative to 'less healthy' products in-store (e.g., through shelf space and strategic placement) and in promotional catalogues

5| **Support** the development of 'free sugar' labelling regulations

COMPANY SCORECARDS
PACKAGED FOOD AND BEVERAGE MANUFACTURERS

Tru Blu Beverages*

16th OUT OF 19 FOOD AND BEVERAGE MANUFACTURERS

9 OVERALL SCORE (OUT OF 100)

Domain	Weighting	Company score
A Corporate strategy	10%	17
B Product formulation	30%	9
C Nutrition labelling	20%	15
D Promotion practices	30%	6
E Product accessibility	5%	0
F Relationships with external groups	5%	0

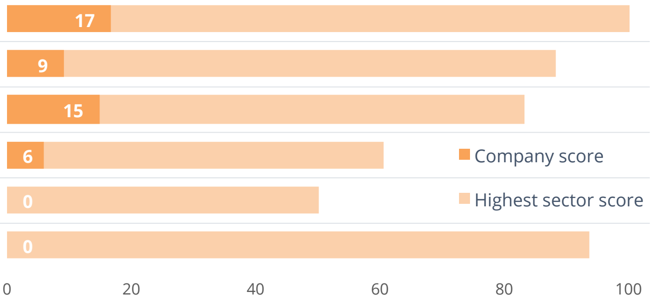

Areas of strength

B| Product formulation - Tru Blu Beverages makes some commitment to reduce sugar in its products

C| Nutrition labelling - Tru Blu Beverages provides comprehensive nutrition information online for its products, both per serve and per 100g

Tru Blu Beverages had little disclosure of its approach to nutrition and health. As such no further areas of strength were identified.

*Assessment based on publicly available information only

Prioritised recommended actions for Tru Blu Beverages

1| **Identify** nutrition and health as a priority focus area for the company, with relevant objectives, targets and appropriate resourcing. Report progress against specific nutrition and health objectives

2| **Develop** specific, time-bound targets for the reduction of free sugar and kilojoule content across the product portfolio. Routinely report on progress in achieving reformulation targets

3| **Publicly commit** to full implementation of the Health Star Rating system across all relevant products, with specific roll-out plan. As part of this commitment, implement interpretive HSR labelling (use of Health Stars, rather than just the energy icon)

4| **Develop** a strategy to increase the proportion of 'healthy' products in the company's portfolio

5| **Commit** to not directly supply any school in Australia with full sugar carbonated beverages or energy drinks

6| **Implement** a policy for reducing promotion of 'less healthy' products/brands that applies to children and adolescents (**up to the age of 18**), across all media channels, and includes all times/events when a large number of children /adolescents are likely to be exposed. Adopt government guidelines for classifying the healthiness of foods

Unilever

3rd OUT OF 19 FOOD AND BEVERAGE MANUFACTURERS

68 OVERALL SCORE (OUT OF 100)

Domain	Weighting
**A** Corporate strategy	10%
**B** Product formulation	30%
**C** Nutrition labelling	20%
**D** Promotion practices	30%
**E** Product accessibility	5%
**F** Relationships with external groups	5%

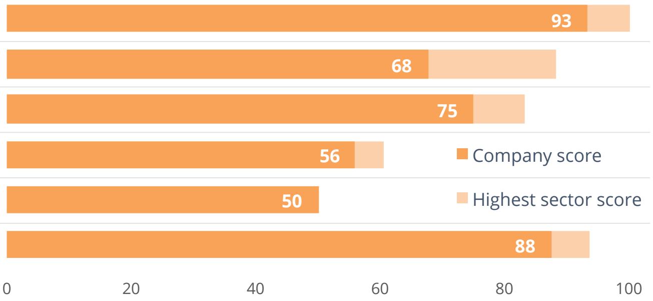

Areas of strength

A| Corporate strategy - Unilever identifies nutrition and health as a priority focus area for the company, communicated through national and global company reporting. The company aligns its nutrition and health agenda with priorities laid out by the WHO and UN Sustainable Development Goals, and regularly reports its progress in achieving specific nutrition and health objectives

B| Product formulation - Unilever has set specific, time-bound targets for the reduction of added sugars, saturated fat, sodium and kilojoules across portions of the company's portfolio, and has reported removal of all artificially produced trans fat from its products

C| Nutrition labelling - Unilever commits to full implementation of the Australian government-endorsed Health Star Rating system across its food and beverage portfolio

E| Product Accessibility - Unilever has set clear, time-bound targets to increase the healthiness of a portion of its portfolio, and commits to address the affordability and availability of its healthy products across the markets in which it operates

Prioritised recommended actions for Unilever

1| **Implement** a strengthened policy for reducing promotion of 'less healthy' products/brands that applies to children and adolescents (**up to the age of 18**), across all media channels, and includes all times/events when a large number of children/adolescents are likely to be exposed. Adopt government guidelines for classifying the healthiness of foods

2| **Develop** specific, time-bound targets for the reduction of nutrients of concern and energy content across all relevant products in the company's portfolio

3| **Participate** in / implement a strategy to adopt relevant recommendations from government-led programs (e.g., Healthy Food Partnership) to improve the healthiness of the food supply

4| **Commit** not to sponsor sporting and community events that are popular with children/families using 'less healthy' products and brands

5| **Eliminate** use of promotion techniques (e.g., cartoon characters, interactive games) with strong appeal to children in relation to 'less healthy' products and brands

6| **Commit** to work with retailers to increase the prominence of healthier products relative to 'less healthy' products in-store (e.g., through shelf space and strategic placement) and in promotional catalogues

ALDI*

3rd OUT OF 4 SUPERMARKETS

11 OVERALL SCORE (OUT OF 100)

Domain	Weighting
**A** Corporate strategy	10%
**B** Product formulation	25%
**C** Nutrition labelling	15%
**D** Promotion practices	25%
**E** Product accessibility	20%
**F** Relationships with external groups	5%

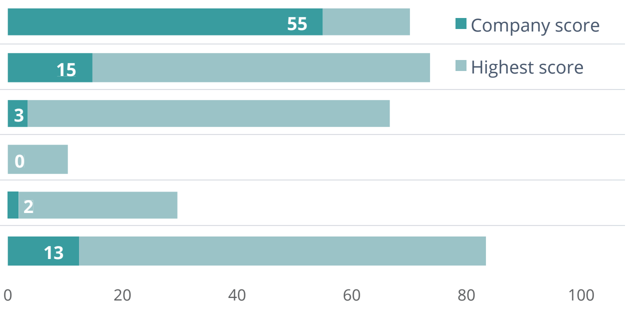

Areas of strength

A| **Corporate strategy** - ALDI makes some commitment to nutrition and health through international Corporate Responsibility reporting. Globally, the company references the United Nations Sustainable Development Goals, and reports against some of its country-specific nutrition objectives and targets

B| **Product formulation** - ALDI globally commits to reduce artificially produced trans fat across its own-brand portfolio to less than 2% of total fat. This commitment applied to all markets in which it operates, including Australia

F| **Relationships with external groups** - ALDI Australia publishes a full list of the philanthropic groups it supports

*Assessment based on publicly available information only

Recommended actions for ALDI

Short-term (next 12 months)

1| **Identify** health and nutrition as a priority focus area for the company at the **national** level, and appropriately resource health and nutrition within the business.

2| **Develop** and publicise specific, time-bound targets for the reduction of sodium, sugar, saturated fat and portion size/energy content in the company's product portfolio. Routinely report on progress in achieving reformulation targets

3| **Commit** to implement the Health Star Rating system across all own-brand products, with specific roll-out plan

4| **Provide** online nutrition information for all products available for sale

5| **Implement** a policy on marketing to children that effectively restricts the exposure of children and adolescents (up to age 18) to promotion of 'less healthy' foods across broadcast and non-broadcast media, using government guidelines for classifying the healthiness of foods. Routinely report on compliance with the policy

Longer term (2-3 years)

6| **Introduce** universal healthy checkouts (with no 'less healthy' products, such as confectionery and sugar-sweetened beverages, on display near registers) across all stores nationally

7| **Commit** to increase the proportion of healthy products (using government guidelines for classifying the healthiness of foods) featured in catalogues and other advertising

8| **Limit** price promotions (e.g., price discounts and 'buy-one-get-one-free' specials) on 'less healthy' products, whilst continuing to increase affordability of healthy products

Coles

2nd OUT OF 4 SUPERMARKETS

40 OVERALL SCORE (OUT OF 100)

Domain	Weighting
**A** Corporate strategy	10%
**B** Product formulation	25%
**C** Nutrition labelling	15%
**D** Promotion practices	25%
**E** Product accessibility	20%
**F** Relationships with external groups	5%

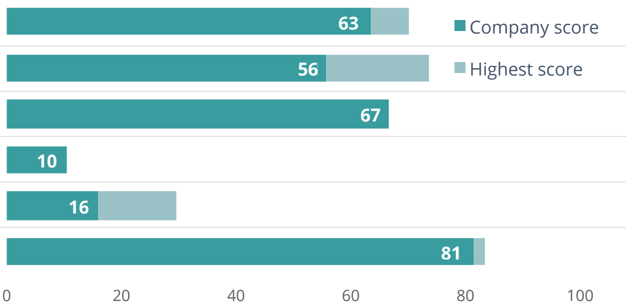

Areas of strength

A| Corporate strategy - Coles highlights nutrition as an area of focus for the company in Wesfarmers Sustainability reporting and reports against some of its progress in achieving nutrition objectives on an annual basis

B| Product formulation - Coles publicly commits to reducing levels of sodium and sugar across a portion of its own-brand product portfolio. The company is involved in the Australian government's Healthy Food Partnership

C| Nutrition labelling - Coles publicly commits to implement the Australian government endorsed HSR system across all products in its own brand portfolio. The company uses the interpretive form of HSR labelling (i.e. star ratings) across all product categories

F| Relationships with external groups - Coles publishes a comprehensive list of external groups related to nutrition and health that it supports, and commits to not make political donations

Recommended actions for Coles

Short-term (next 12 months)

1| **Elevate** health and nutrition to a higher priority focus area within the business, and ensure adequate resources are allocated to this area.

2| **Develop** and publicise specific, time-bound targets for the reduction of sodium, sugar, saturated fat and portion size/energy content across all relevant products in the company's own-brand portfolio. Routinely report on progress in achieving reformulation targets

3| **Implement** a marketing to children policy that effectively restricts the exposure of children and adolescents (up to age 18) to promotion of 'less healthy' foods across broadcast and non-broadcast media, using government guidelines for classifying the healthiness of foods. Routinely report on compliance with the policy

4| **Expand** online nutrition information to include all products available for sale

Longer term (2-3 years)

5| **Introduce** universal healthy checkouts (with no 'less healthy' products, such as confectionery and sugar-sweetened beverages, on display near registers) across all stores nationally

6| **Commit** to increase the proportion of healthy products (using government guidelines for classifying the healthiness of foods) featured in catalogues and other advertising

7| **Limit** price promotions (e.g., price discounts and 'buy-one-get-one-free' specials) on 'less healthy' products, whilst continuing to increase affordability of healthy products

8| **Link** rewards through loyalty programs to healthier products

9| **Increase** the proportion of healthy products displayed in high-traffic areas (e.g., end-of-aisle displays)

IGA

4th OUT OF 4 SUPERMARKETS

8 OVERALL SCORE (OUT OF 100)

Domain	Weighting
**A** Corporate strategy	10%
**B** Product formulation	25%
**C** Nutrition labelling	15%
**D** Promotion practices	25%
**E** Product accessibility	20%
**F** Relationships with external groups	5%

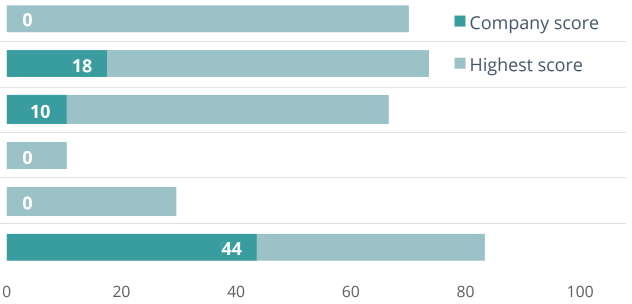

Areas of strength

- IGA had little public disclosure of its approach to nutrition and health. As such, no clear areas of strength were identified
- Selected IGA stores in Victoria are involved in a trial, *Eat Well @ IGA*, of interventions designed to improve the healthiness of customer purchases, in partnership with VicHealth, the City of Greater Bendigo and Deakin University

Recommended actions for IGA

Short-term (next 12 months)

1| **Identify** health and nutrition as a priority focus area for the company, articulated through the company's mission statement and sustainability reporting. Centralise the company's nutrition and health approach so that it applies to all IGA's nationally, and appropriately resource nutrition and health within the business.

2| **Develop** and publicise specific, time-bound targets for the reduction of sodium, sugar, saturated fat and portion size/energy content in the company's product portfolio. Routinely report on progress in achieving reformulation targets

3| **Commit** to implement the Health Star Rating system across all own-brand products in all stores nationally and develop a time-bound roll-out plan

4| **Provide** online nutrition information for all products available for sale

5| **Implement** a policy on marketing to children that effectively restricts the exposure of children and adolescents (up to age 18) to promotion of 'less healthy' foods across broadcast and non-broadcast media, using government guidelines classifying the healthiness of foods. Routinely report on compliance with the policy

Longer term (2-3 years)

6| **Introduce** universal healthy checkouts (with no 'less healthy' products, such as confectionery and sugar-sweetened beverages, on display near registers) across all stores nationally

7| **Commit** to increase the proportion of healthy products (using government guidelines for classifying the healthiness of foods) featured in catalogues and other advertising

8| **Limit** price promotions (e.g., price discounts and 'buy-one-get-one-free' specials) on 'less healthy' products across all IGA stores nationally

Woolworths

1st OUT OF 4 SUPERMARKETS

46 OVERALL SCORE (OUT OF 100)

Domain	Weighting
**A** Corporate strategy	10%
**B** Product formulation	25%
**C** Nutrition labelling	15%
**D** Promotion practices	25%
**E** Product accessibility	20%
**F** Relationships with external groups	5%

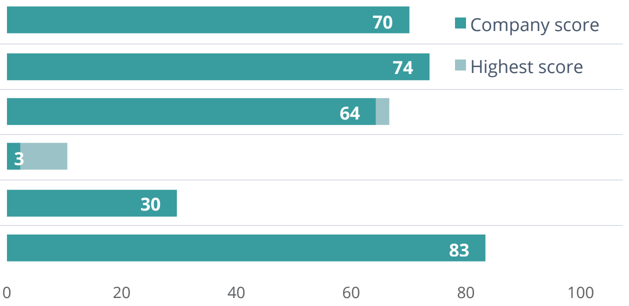

Areas of strength

A| **Corporate strategy** - Woolworths identifies nutrition and health as a priority, communicated through the company's overarching mission statement and corporate reporting. Woolworths' approach to nutrition and health is reported against annually, and references priorities laid out by the United Nations Sustainable Development Goals

B| **Product formulation** - Woolworths commits to reducing levels of sugar, sodium and saturated fat across portions of its own-brand portfolio. Woolworths published its support for the Australian government's Healthy Food Partnership in company reporting

C| **Nutrition labelling** - Woolworths publicly commits to implement the Australian government-endorsed HSR system across all products in its own-brand portfolio. The company reports progress on implementing HSR labelling, and uses the interpretive form (i.e. star ratings) across all product categories. Woolworths provides comprehensive nutrition information online for the majority of products available for sale

E| **Product accessibility** - Woolworths provides 'free fruit for kids' in all stores nationally

Recommended actions for Woolworths

Short-term (next 12 months)

1| **Develop** and publicise specific, time-bound targets for the reduction of sodium, sugar, saturated fat and portion size/energy content across **all** product categories in the company's own-brand portfolio. Routinely report on progress in achieving reformulation targets

2| **Implement** a policy on marketing to children that effectively restricts the exposure of children and adolescents (up to age 18) to promotion of 'less healthy' foods across broadcast and non-broadcast media, using government guidelines for classifying the healthiness of foods. Routinely report on compliance with the policy

Longer term (2-3 years)

3| **Introduce** universal healthy checkouts (with no 'less healthy' products, such as confectionery and sugar-sweetened beverages, on display near registers) across all stores nationally

4| **Commit** to increase the proportion of healthy products (using government guidelines for classifying the healthiness of foods) featured in catalogues and other advertising

5| **Limit** price promotions (e.g., price discounts and 'buy-one-get-one-free' specials) on 'less healthy' products, whilst continuing to increase affordability of healthy products

6| **Link** rewards through loyalty programs to healthier products

7| **Increase** the proportion of healthy products displayed in high-traffic areas (e.g., end-of-aisle displays)

COMPANY SCORECARDS
QUICK SERVICE RESTAURANTS

Chicken Treat*

7th OUT OF 11 QUICK SERVICE RESTAURANTS

14 OVERALL SCORE (OUT OF 100)

Domain	Weighting
**A** Corporate strategy	10%
**B** Product formulation	25%
**C** Nutrition labelling	15%
**D** Promotion practices	25%
**E** Product accessibility	20%
**F** Relationships with external groups	5%

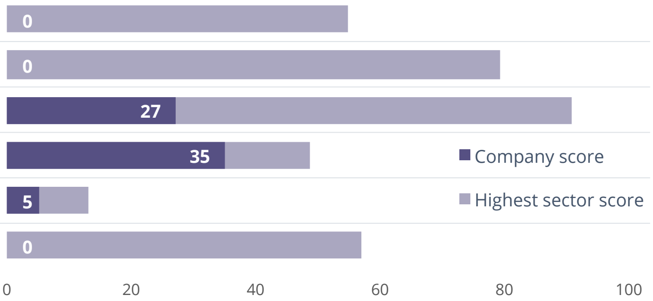

Areas of strength

C| Nutrition labelling - Chicken Treat provides comprehensive nutrition information online

D| Promotion practices – Chicken Treat is a signatory to the AFGC's 'QSR Initiative for Responsible Advertising and Marketing to Children'

- Chicken Treat had little disclosure of its approach to health and nutrition, as such, no further areas of strength were identified

*Assessment based on publicly available information only

Prioritised recommended actions for Chicken Treat

1| **Identify** health and nutrition as a priority focus area for the company, with relevant objectives, targets and appropriate resourcing. Report progress against health and nutrition objectives and targets

2| **Develop** specific, measurable targets for the reduction of added sugar, saturated fat, sodium and kilojoule content across relevant menu items. Routinely report against progress in achieving reformulation commitments

3| **Participate in** / implement a strategy to adopt relevant recommendations from government-led initiatives (e.g., Healthy Food Partnership) to improve the healthiness of the food supply

4| **Implement** a policy for reducing the exposure of children and adolescents (up to the age of 18) to promotion of 'less healthy' foods/brands that applies across all media channels, and includes all times/events when a large number of children/adolescents are likely to be exposed

5| **Commit** to make healthier meal options (e.g., healthier sides and drinks) the default option, particularly as part of children's meals

6| **Introduce** a pricing strategy that positions healthier menu items at a similar or lower price to 'less healthy' equivalents, and **restrict** price promotions and value deal incentives that include 'less healthy' side and/or drink items

7| **Commit** to not sponsor sporting and community events that are popular with children and families

Domino's Pizza*

11th **OUT OF 11 QUICK SERVICE RESTAURANTS**

3 **OVERALL SCORE** (OUT OF 100)

Domain	Weighting
**A** Corporate strategy	10%
**B** Product formulation	25%
**C** Nutrition labelling	15%
**D** Promotion practices	25%
**E** Product accessibility	20%
**F** Relationships with external groups	5%

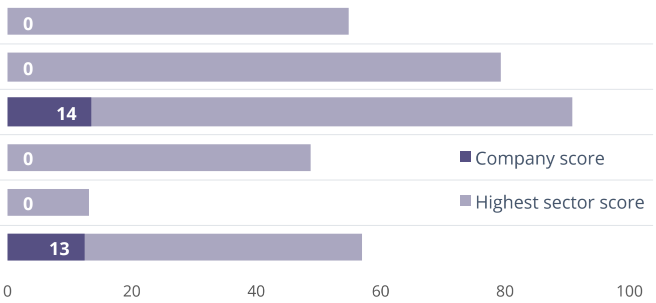

Areas of strength

C| **Nutrition labelling** - Domino's Pizza provides comprehensive nutrition information online

- Domino's Pizza had little disclosure of its approach to health and nutrition. As such, no further areas of strength were identified

*Assessment based on publicly available information only

Prioritised recommended actions for Domino's Pizza

1| **Identify** health and nutrition as a priority focus area for the company, with relevant objectives, targets and appropriate resourcing. Report progress against health and nutrition objectives and targets

2| **Develop** specific, measurable targets for the reduction of added sugar, saturated fat, sodium and kilojoule content across relevant menu items. Routinely report against progress in achieving reformulation commitments

3| **Participate in** / implement a strategy to adopt relevant recommendations from government-led initiatives (e.g., Healthy Food Partnership) to improve the healthiness of the food supply

4| **Implement** a policy for reducing the exposure of children and adolescents (up to the age of 18) to promotion of 'less healthy' foods/brands that applies across all media channels, and includes all times/events when a large number of children/adolescents are likely to be exposed.

5| **Commit** to not sponsor sporting and community events that are popular with children and families

6| **Commit** to make healthier meal options (e.g., healthier sides and drinks) the default option

7| **Introduce** a pricing strategy that positions healthier menu items at a similar or lower price to 'less healthy' equivalents, and **restrict** price promotions and value deal incentives that include 'less healthy' side and/or drink items

Grill'd*

10th OUT OF 11 QUICK SERVICE RESTAURANTS

10 OVERALL SCORE (OUT OF 100)

Domain	Weighting
**A** Corporate strategy	10%
**B** Product formulation	25%
**C** Nutrition labelling	15%
**D** Promotion practices	25%
**E** Product accessibility	20%
**F** Relationships with external groups	5%

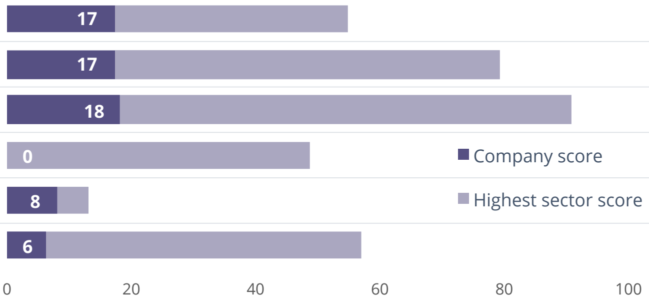

Areas of strength

A| Corporate strategy - Grill'd identifies health as a focus area for the company

B| Product formulation - Grill'd commits to using healthier (non-hydrogenated) frying oils, and has reduced sugar in a number of menu items

C| Nutrition labelling – Grill'd provides comprehensive nutrition information online

- Grill'd had little disclosure of its approach to health and nutrition. As such, no further areas of strength were identified

Prioritised recommended actions for Grill'd

1| Publicly **communicate** the company's approach to nutrition and health in corporate reporting, setting clear targets and objectives that are routinely reported against

2| **Develop** specific, measurable targets for the reduction of saturated fat, sodium and kilojoule content across relevant menu items. Routinely report against progress in achieving reformulation commitments

3| **Participate in** / implement a strategy to adopt relevant recommendations from government-led initiatives (e.g., Healthy Food Partnership) to improve the healthiness of the food supply

4| **Publish** a formal policy related to reducing the exposure of children and adolescents (up to the age of 18) to promotion of 'less healthy' foods/ brands that applies across all media channels, and includes all times/events when a large number of children/adolescents are likely to be exposed.

5| **Commit** to make healthier meal options (e.g., healthier sides and drinks) the default option for children's meals

6| **Publicly** commit to implement kilojoule labelling on menu boards across all states/ territories, and **support** the development of standardised interpretive nutrition labelling (e.g., using health stars or colour-coding) for menu boards

*Assessment based on publicly available information only

Hungry Jack's*

5th OUT OF 11 QUICK SERVICE RESTAURANTS

28 OVERALL SCORE (OUT OF 100)

Domain	Weighting
**A** Corporate strategy	10%
**B** Product formulation	25%
**C** Nutrition labelling	15%
**D** Promotion practices	25%
**E** Product accessibility	20%
**F** Relationships with external groups	5%

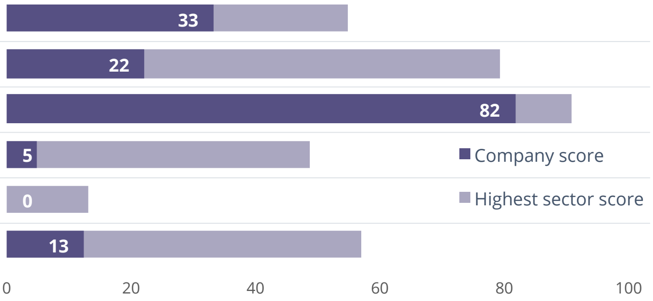

Areas of strength

A| **Corporate strategy** – Hungry Jack's makes some commitment to nutrition in corporate social responsibility reporting

B| **Product formulation** – Hungry Jack's makes a general commitment to reduce levels of saturated fat, added sugar and sodium across some of its menu items

C| **Nutrition labelling** – Hungry Jack's publicly commits to implement kilojoule menu board labelling in all states/territories, and provides comprehensive nutrition information in-store and online

D| **Promotion practices** – Hungry Jack's is a signatory to the AFGC's 'QSR Initiative for Responsible Advertising and Marketing to Children'

*Assessment based on publicly available information only

Prioritised recommended actions for Hungry Jack's

1| **Identify** health and nutrition as a priority focus area for the company, with relevant objectives, targets and appropriate resourcing. Report progress against health and nutrition objectives and targets

2| **Develop** specific, measurable targets for the reduction of added sugar, saturated fat, sodium and kilojoule content across relevant menu items. Routinely report against progress in achieving reformulation commitments

3| **Participate in** / implement a strategy to adopt relevant recommendations from government-led initiatives (e.g., Healthy Food Partnership) to improve the healthiness of the food supply

4| **Implement** a policy for reducing the exposure of children and adolescents (up to the age of 18) to promotion of 'less healthy' foods/brands that applies across all media channels, and includes all times/events when a large number of children/adolescents are likely to be exposed.

5| **Commit** to not sponsor sporting and community events that are popular with children and families

6| **Commit** to make healthier meal options (e.g., healthier sides and drinks) the default option, particularly as part of children's meals

7| **Introduce** a pricing strategy that positions healthier menu items at a similar or lower price to 'less healthy' equivalents, and **restrict** price promotions and value deal incentives that include 'less healthy' side and/or drink items

KFC*

3rd OUT OF 11 QUICK SERVICE RESTAURANTS

41 OVERALL SCORE (OUT OF 100)

Domain	Weighting
**A** Corporate strategy	10%
**B** Product formulation	25%
**C** Nutrition labelling	15%
**D** Promotion practices	25%
**E** Product accessibility	20%
**F** Relationships with external groups	5%

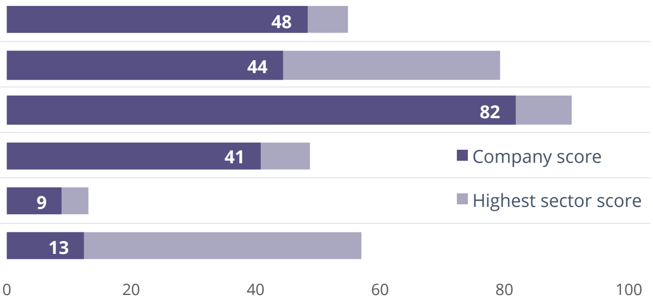

Areas of strength

A| Corporate strategy - KFC outlines its approach to nutrition and health in corporate reporting at the national and global level. The company has some national reporting against its commitment to nutrition-related issues

B| Product formulation – KFC reports against some of its progress to reduce sodium, fat and trans fat, and makes a general commitment to reduce sugar. The company commits to introduce a healthier range of menu items and is a participant in the Australian government's Healthy Food Partnership

C| Nutrition labelling – KFC publicly commits to implement kilojoule menu board labelling across all states/territories, and provides comprehensive nutrition information in-store and online

D| Promotion practices – KFC publicly commits to not offer toys in its children's meals, and is a signatory to the AFGC's 'QSR Initiative for Responsible Advertising and Marketing to Children'

*Assessment based on publicly available information only

Prioritised recommended actions for KFC

1| **Introduce** regular reporting against specific nutrition and health objectives at the national level, referring to relevant international priorities (e.g., as articulated in the UN Sustainable Development Goals or the WHO Global NCD Action Plan)

2| **Set** specific, measurable targets for the reduction of added sugar, saturated fat, sodium and kilojoule content across relevant menu items. Routinely report against progress in achieving reformulation commitments

3| **Implement** a policy for reducing the exposure of children and adolescents (up to the age of 18) to promotion of 'less healthy' foods/brands that applies across all media channels, and includes all times/events when a large number of children/adolescents are likely to be exposed. Routinely report on compliance with the policy

4| **Commit** to not sponsor sporting and community events that are popular with children and families

5| **Commit** to make healthier meal options (e.g., healthier sides and drinks) the default option, particularly as part of children's meals

6| **Introduce** a pricing strategy that positions healthier menu items at a similar or lower price to 'less healthy' equivalents, and **restrict** price promotions and value deal incentives that include 'less healthy' side and/or drink items

7| **Support** the development of standardised interpretive nutrition labelling (e.g., using health stars or colour-coding) for menu boards

McDonald's*

2nd OUT OF 11 QUICK SERVICE RESTAURANTS

42 OVERALL SCORE (OUT OF 100)

Domain	Weighting
**A** Corporate strategy	10%
**B** Product formulation	25%
**C** Nutrition labelling	15%
**D** Promotion practices	25%
**E** Product accessibility	20%
**F** Relationships with external groups	5%

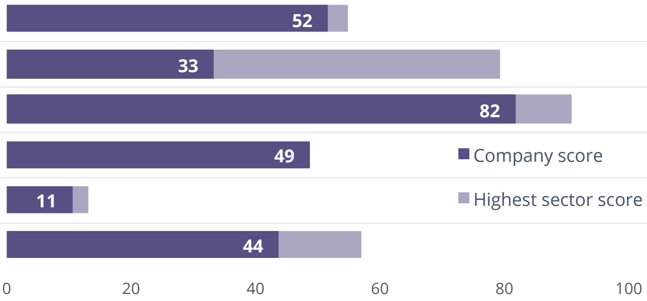

Areas of strength

A| **Corporate strategy** - McDonald's identifies nutrition and health related issues in corporate reporting at the global level, and to some extent nationally

B| **Product formulation** – McDonald's reports against some of its progress to reduce sodium, fat, and sugar, and commits to use frying oils that are virtually free from trans fats

C| **Nutrition labelling** – McDonald's publicly commits to implement kilojoule menu board labelling across all states/territories, and provides comprehensive nutrition information in-store (including on product packaging) and online

D| **Promotion practices** – McDonald's is a signatory to the AFGC's 'QSR Initiative for Responsible Advertising and Marketing to Children'

*Assessment based on publicly available information only

Prioritised recommended actions for McDonald's

1| **Introduce** regular reporting against specific nutrition and health objectives at the national level, referring to relevant international priorities (e.g., as articulated in the UN Sustainable Development Goals or the WHO Global NCD Action Plan)

2| **Set** measurable targets for the reduction of salt, added sugars, saturated fat, kilojoule content and trans fat across all relevant menu items. Routinely report on progress in achieving reformulation commitments.

3| **Implement** a policy for reducing the exposure of children and adolescents (up to the age of 18) to promotion of 'less healthy' foods/brands that applies across all media channels, and includes all times/events when a large number of children/adolescents are likely to be exposed. Routinely report on compliance with the policy

4| **Eliminate** use of promotion techniques (e.g., cartoon characters, interactive games, toys in children's meals) with strong appeal to children

5| **Commit** to not sponsor sporting and community events that are popular with children and families

6| **Commit** to make healthier meal options (e.g., healthier sides and drinks) the default option, particularly as part of children's meals

7| **Introduce** a pricing strategy that positions healthier menu items at a similar or lower price to 'less healthy' equivalents, and **restrict** price promotions and value deal incentives that include 'less healthy' side and/or drink items

Nando's

4th OUT OF 11 QUICK SERVICE RESTAURANTS

31 OVERALL SCORE (OUT OF 100)

Domain	Weighting
**A** Corporate strategy	10%
**B** Product formulation	25%
**C** Nutrition labelling	15%
**D** Promotion practices	25%
**E** Product accessibility	20%
**F** Relationships with external groups	5%

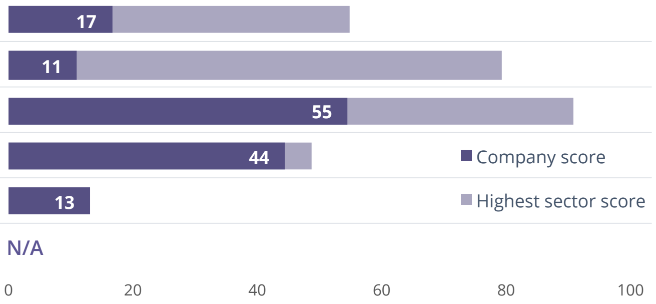

Areas of strength

A| **Corporate strategy** - Nando's has some internal commitment to health and nutrition

C| **Nutrition labelling** – Nando's implements kilojoule menu board labelling in all states/ territories, and provides nutrition information online

E| **Product accessibility** – Nando's offers healthier side and drink options as part of meal deals and children's meals

Prioritised recommended actions for Nando's

1| **Identify** health and nutrition as a priority focus area for the company, with relevant objectives, targets and appropriate resourcing. Report progress against health and nutrition objectives and targets

2| **Develop** and publicise specific, measurable targets for the reduction of added sugar, saturated fat, sodium and kilojoule content across menu items, where applicable. Routinely report against progress in achieving reformulation commitments

3| **Participate in** / implement a strategy to adopt relevant recommendations from government-led initiatives (e.g., Healthy Food Partnership) to improve the healthiness of the food supply

4| **Implement** a formal policy for reducing the exposure of children and adolescents (up to the age of 18) to promotion of 'less healthy' foods that applies across all media channels, and includes all times/events when a large number of children/adolescents are likely to be exposed. Routinely report on compliance with the policy

5| **Commit** to make healthier meal options (e.g., healthier sides and drinks) the default option, particularly as part of children's meals

6| **Introduce** a pricing strategy that positions healthier menu items at a similar or lower price to 'less healthy' equivalents, and **restrict** price promotions and value deal incentives that include 'less healthy' side and/or drink items

7| **Support** the development of standardised interpretive nutrition labelling (e.g., using health stars or colour-coding) for menu boards

Oporto*

9th OUT OF 11 QUICK SERVICE RESTAURANTS

11 OVERALL SCORE (OUT OF 100)

Domain	Weighting
**A** Corporate strategy	10%
**B** Product formulation	25%
**C** Nutrition labelling	15%
**D** Promotion practices	25%
**E** Product accessibility	20%
**F** Relationships with external groups	5%

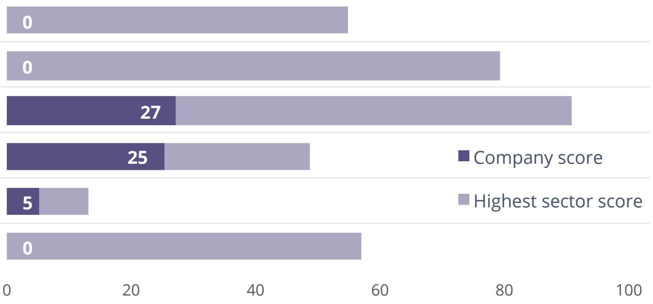

Areas of strength

C | **Nutrition labelling** - Oporto provides comprehensive nutrition information online

D | **Promotion practices** – Oporto is a signatory to the AFGC's 'QSR Initiative for Responsible Advertising and Marketing to Children'

- Oporto had little disclosure of its approach to health and nutrition. As such, no further areas of strength were identified

*Assessment based on publicly available information only

Prioritised recommended actions for Oporto

1 | **Identify** health and nutrition as a priority focus area for the company, with relevant objectives, targets and appropriate resourcing. Report progress against health and nutrition objectives and targets

2 | **Develop** specific, measurable targets for the reduction of added sugar, saturated fat, sodium and kilojoule content across relevant menu items. Routinely report against progress in achieving reformulation commitments

3 | **Participate in** / implement a strategy to adopt relevant recommendations from government-led initiatives (e.g., Healthy Food Partnership) to improve the healthiness of the food supply

4 | **Implement** a policy for reducing the exposure of children and adolescents (up to the age of 18) to promotion of 'less healthy' foods/brands that applies across all media channels, and includes all times/events when a large number of children/adolescents are likely to be exposed

5 | **Commit** to make healthier meal options (e.g., healthier sides and drinks) the default option, particularly as part of children's meals

6 | **Introduce** a pricing strategy that positions healthier menu items at a similar or lower price to 'less healthy' equivalents, and **restrict** price promotions and value deal incentives that include 'less healthy' side and/or drink items

7 | **Commit** to not sponsor sporting and community events that are popular with children and families

Pizza Hut*

6th OUT OF 11 QUICK SERVICE RESTAURANTS

27 OVERALL SCORE (OUT OF 100)

Domain	Weighting
**A** Corporate strategy	10%
**B** Product formulation	25%
**C** Nutrition labelling	15%
**D** Promotion practices	25%
**E** Product accessibility	20%
**F** Relationships with external groups	5%

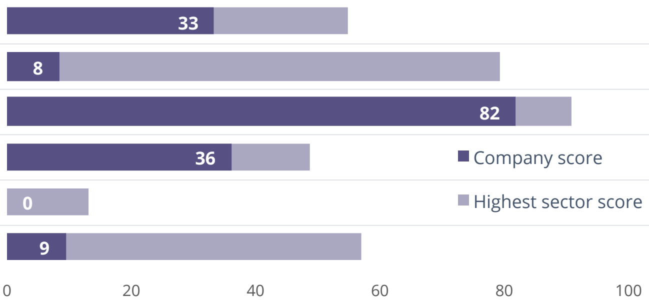

Areas of strength

A| **Corporate strategy** – Pizza Hut commits to reduce sodium across a number of menu items

C| **Nutrition labelling** – Pizza Hut publicly commits to implement kilojoule menu board labelling in all states/territories, and provides comprehensive nutrition information in-store and online

D| **Promotion practices** – Pizza Hut is a signatory to the AFGC's 'QSR Initiative for Responsible Advertising and Marketing to Children'

*Assessment based on publicly available information only

Prioritised recommended actions for Pizza Hut

1| **Identify** health and nutrition as a priority focus area for the company, with relevant objectives, targets and appropriate resourcing. Report progress against health and nutrition objectives and targets

2| **Develop** specific, measurable targets for the reduction of added sugar, saturated fat, sodium and kilojoule content across relevant menu items. Routinely report against progress in achieving reformulation commitments

3| **Participate in** / implement a strategy to adopt relevant recommendations from government-led initiatives (e.g., Healthy Food Partnership) to improve the healthiness of the food supply

4| **Implement** a policy for reducing the exposure of children and adolescents (up to the age of 18) to promotion of 'less healthy' foods/brands that applies across all media channels, and includes all times/events when a large number of children/adolescents are likely to be exposed.

5| **Commit** to not sponsor sporting and community events that are popular with children and families

6| **Commit** to make healthier meal options (e.g., healthier sides and drinks) the default option

7| **Introduce** a pricing strategy that positions healthier menu items at a similar or lower price to 'less healthy' equivalents, and **restrict** price promotions and value deal incentives that include 'less healthy' side and/or drink items

Red Rooster*

8th OUT OF 11 QUICK SERVICE RESTAURANTS

12 OVERALL SCORE (OUT OF 100)

Domain	Weighting
**A** Corporate strategy	10%
**B** Product formulation	25%
**C** Nutrition labelling	15%
**D** Promotion practices	25%
**E** Product accessibility	20%
**F** Relationships with external groups	5%

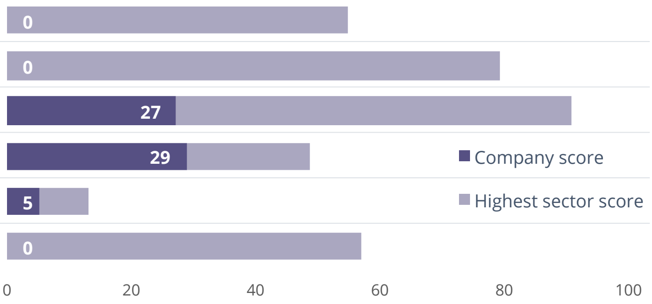

Areas of strength

C| **Nutrition labelling** - Red Rooster provides comprehensive nutrition information online

D| **Promotion practices** – Red Rooster is a signatory to the AFGC's 'QSR Initiative for Responsible Advertising and Marketing to Children'

- Red Rooster had little disclosure of its approach to health and nutrition. As such, no further areas of strength were identified

*Assessment based on publicly available information only

Prioritised recommended actions for Red Rooster

1| **Identify** health and nutrition as a priority focus area for the company, with relevant objectives, targets and appropriate resourcing. Report progress against health and nutrition objectives and targets

2| **Develop** specific, measurable targets for the reduction of added sugar, saturated fat, sodium and kilojoule content across relevant menu items. Routinely report against progress in achieving reformulation commitments

3| **Participate in** / implement a strategy to adopt relevant recommendations from government-led initiatives (e.g., Healthy Food Partnership) to improve the healthiness of the food supply

4| **Implement** a policy for reducing the exposure of children and adolescents (up to the age of 18) to promotion of 'less healthy' foods/brands that applies across all media channels, and includes all times/events when a large number of children/adolescents are likely to be exposed

5| **Commit** to make healthier meal options (e.g., healthier sides and drinks) the default option, particularly as part of children's meals

6| **Introduce** a pricing strategy that positions healthier menu items at a similar or lower price to 'less healthy' equivalents, and **restrict** price promotions and value deal incentives that include 'less healthy' side and/or drink items

7| **Commit** to not sponsor sporting and community events that are popular with children and families

Subway

1st OUT OF 11 QUICK SERVICE RESTAURANTS

48 OVERALL SCORE (OUT OF 100)

Domain	Weighting
**A** Corporate strategy	10%
**B** Product formulation	25%
**C** Nutrition labelling	15%
**D** Promotion practices	25%
**E** Product accessibility	20%
**F** Relationships with external groups	5%

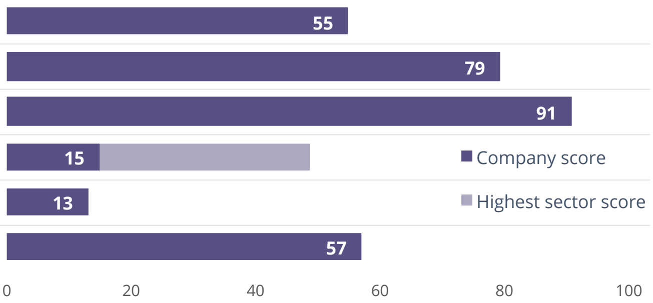

Areas of strength

A| **Corporate strategy** - Subway identifies nutrition and nutritious food as a key part of its business strategy. Globally, the company has some reporting against its progress in achieving health and nutrition objectives

B| **Product formulation** – Subway has reformulated its menu items to reduce levels of sodium and fat, and has eliminated artificially produced trans fat from its products. Subway commits to limit kilojoule content in several menu items. Subway is a nominated participant of the Australian government's Healthy Food Partnership

C| **Nutrition labelling** – Subway publicly commits to implement kilojoule menu board labelling across all states/territories, and provides comprehensive nutrition information in-store and online

E| **Product accessibility** – Subway offers only healthier side and drink items as the default in its children's meals

Prioritised recommended actions for Subway

1| Further **communicate** the company's approach to nutrition and health at the national level, setting clear objectives and targets that are routinely reported against

2| **Publicise** specific targets to reduce levels of added sugar, kilojoules and saturated fat across menu items, where applicable

3| **Extend** the company's internal commitment by implementing a formal policy for reducing the exposure of children and adolescents (up to the age of 18) to promotion of 'less healthy' foods that applies across all media channels, and includes all times/events when a large number of children/adolescents are likely to be exposed. Routinely report on compliance with the policy

4| **Commit** to phase out free refills on soft drinks, in stores where this is applicable

5| **Introduce** a pricing strategy that positions healthier menu items at a similar or lower price to 'less healthy' equivalents, and **restrict** price promotions and value deal incentives that include 'less healthy' side and/or drink items

6| **Support** the development of standardised interpretive nutrition labelling (e.g., using health stars or colour-coding) for menu boards
