# Supplementary material for: Benchmarking the Nutrition-Related Policies and Commitments of Major Food Companies in Australia, 2018
Source: Int J Environ Res Public Health. 2020 Aug 22;17(17):6118. doi: 10.3390/ijerph17176118 (PMC7504100; doi:10.3390/ijerph17176118)
Supplement: Supplementary file 1 [file ijerph-17-06118-s001.zip › Table S3 - BIA-Obesity Australia 2018 IJERPH.docx]

Supplementary material

Table S3: *BIA-Obesity Australia 2018* assessment tool for quick service restaurants (R)

| **Indicator ID** | **Domain and indicator** | | **Scoring** | |
| --- | --- | --- | --- | --- |
| **R-STRAT** | **CORPORATE STRATEGY** | | **MAXIMUM TOTAL POINTS = 30 (PRE-WEIGHTING)**  **WEIGHTING APPLIED = 10** | |
| **R-STRAT1** | Does the company have an overarching commitment to improving population nutrition and health articulated in strategic documents (e.g., corporate strategy document, corporate responsibility reports)? | | 10: Yes, a specific national-level (Australia-specific) commitment to improving population nutrition and health, publicly available in strategic documents  7.5: Yes, a specific global-level (not Australia-specific) commitment to improving population nutrition and health, publicly available in strategic documents  5: Yes, a national- or global-level commitment, but not publicly-available, OR general reference to nutrition and health as part of general corporate strategy  0: No clear commitments to improving population nutrition and health | |
| **R-STRAT2** | Does the company’s commitment to improving population nutrition and health (where it exists) include specific objectives and targets for obesity and NCDs? | | (Can be multiple, max of 10 points)  2: Contains specific, measurable, achievable, relevant and time bound (SMART) objectives and targets  2: Recognition or reference to relevant priorities set out in the WHO Global Action Plan for the Prevention and Control of NCDs 2013-2020, Sustainable Development Goals, or the WHO Report on Ending Childhood Obesity  2: Recognition or reference to relevant priorities in national government policy documents relating to population nutrition and obesity/NCD prevention  2: Comprehensive in nature (e.g., includes three or more domains in this document, such as formulation, marketing and labelling)  2: Key Performance Indicators (KPIs) (and/or remuneration) of senior managers linked to nutrition strategy/policy/targets  1 point if specified globally but not nationally | |
| **R-STRAT3** | Does the company regularly publish details of its approach to population nutrition and health related to obesity and NCDs? | | 10: Annual, publicly available reports including reporting against objectives and targets, a clear outlook of future plans and challenges, external verification / review, and specifically relate to the country in question  7.5: Annual, publicly available global reports that are not clear whether or not they include the country in question  5: Annual reports including some of the relevant information, available upon request  2.5: Irregular reporting  0: None published | |
|  | | | | |
| **R-FORM** | **PRODUCT FORMULATION** | | **MAXIMUM TOTAL POINTS = 90 (PRE-WEIGHTING)**  **WEIGHTING APPLIED = 25** | |
| **R-FORM1** | Does the company publish a comprehensive set of commitments or objectives related to new product development and reformulating its existing products with respect to the nutrients of concern and energy (salt, saturated fats, trans fats, added sugar and kilojoules)? | | 10: Yes, specific national-level commitments/objectives that are publicly available or specific global commitments/objectives that include specific reference to the country or market in question  7.5: Yes, specific global commitments/objectives that could specifically apply to the country in question that are publicly available  5: Has specific national-level commitments/objectives, but not publicly available  2.5: Has national or global-level commitments/objectives in this area that are available publicly, but these commitments/objectives are vague and non-specific OR has global commitments/objectives but not publicly available  0: No commitment/ no policy information available to the research team | |
| **R-FORM2** | Is the company a signatory to national and/or global industry initiatives on product reformulation or do they commit to national voluntary government programs on product reformulation? | | 5: Yes, and noted on company website / annual reports  2.5 Yes, but not noted on company website / annual reports (e.g. government/ NGO/ industry organisation’s website or disclosed directly to INFORMAS)  0: No | |
|  | ***Salt/sodium targets*** | | | |
| **R-FORM3.1** | Has the company set a target/targets or provided detailed evidence of having taken substantive action to reduce/reach lower levels of salt/sodium in products? | | (Can be multiple)  5: Set SMART target or provided detailed evidence of having taken substantive action for children’s meals, published (2.5 if not published, or not SMART)  5: Set SMART target or provided detailed evidence of having taken substantive action for other relevant products/sub-categories, published (2.5 if not published, or not SMART)  0: No target / no information | |
| **R-FORM3.2** | When is the baseline year and target year?  What are the targets? | | [Information only, not to be scored] | |
|  | ***Trans fats targets*** | | | |
| **R-FORM4.1** | Has the company set a target/targets or provided detailed evidence of having taken substantive action to reduce artificial trans fat added to products during the manufacturing process? | | 10: Set a target or provided detailed evidence of having taken substantive action to eliminate artificial trans fat in all relevant categories/subcategories, published  5: Set a target or provided detailed evidence of having taken substantive action to eliminate/reduce in some relevant products/sub-categories / not published  0: No target / no information | |
| **R-FORM4.2** | When is the baseline year and target year?  What are the targets? | | [Information only, for evidence document, not to be scored] | |
| **R-FORM4.3** | Does the restaurant have commitments or provided detailed evidence of having taken substantive action to improving the healthiness of oils used in frying foods / frying practices? (if applicable) | | 5: Does not fry foods, or specifically commits to using non-hydrogenated vegetable oils  0: No commitments / no information available to the research team | |
|  | ***Saturated fats targets*** | | | |
| **R-FORM5.1** | Has the company set a target/targets or provided detailed evidence of having taken substantive action to reduce/reach lower levels of saturated fats | | (Can be multiple)  5: Set SMART target or provided detailed evidence of having taken substantive action for children’s meals, published (2.5 if not published, or not SMART)  5: Set SMART target or provided detailed evidence of having taken substantive action for other relevant products/sub-categories, published (2.5 if not published, or not SMART)  0: No target / no information | |
| **R-FORM5.2** | When is the baseline year and target year?  What are the targets? | | [Information only, for evidence document, not to be scored] | |
|  | ***Added sugars targets*** | | | |
| **R-FORM6.1** | Has the company set a target/targets or provided detailed evidence of having taken substantive action to reduce/reach lower levels of added sugars? | | (Can be multiple)  5: Set SMART target or provided detailed evidence of having taken substantive action for children’s meals, published (2.5 if not published, or not SMART)  5: Set SMART target or provided detailed evidence of having taken substantive action for other relevant products/sub-categories, published (2.5 if not published, or not SMART)  0: No target / no information | |
| **R-FORM6.2** | When is the baseline year and target year?  What are the targets? | | [Information only, for evidence document, not to be scored] | |
|  | ***Portion size (energy content) targets*** | | | |
| **R-FORM7.1** | Has the company set a target/targets or provided detailed evidence of having taken substantive action to reduce the portion size / energy content? | | (Can be multiple)  5: Set SMART target or provided detailed evidence of having taken substantive action for children’s meals, published (2.5 if not published, or not SMART)  5: Set SMART target or provided detailed evidence of having taken substantive action for other relevant products/sub-categories, published (2.5 if not published, or not SMART)  0: No target / no information | |
| **R-FORM7.2** | When is the baseline year and target year?  What are the targets? | | [Information only, for evidence document, not to be scored] | |
|  | ***Classification system*** | |  | |
| **R-FORM8.1** | What system / criteria (e.g., product classification system or nutrient profiling system) does the company use for the purposes of product development / reformulation? | | 10: Uses government guidelines (e.g. health star rating, health claims criteria)  7.5: Publicly available system, developed in consultation with experts and in line with government guidelines, published in peer reviewed literature  5: Publicly available system, developed in consultation with experts and in line with government guidelines (not published in peer reviewed literature)  2.5: Publicly available system with no details of development/alignment with government guidelines OR not publicly available but developed in consultation with experts and aligned with government guidelines  0: No information / poor alignment / does not have a system | |
| **R-FORM8.2** | If a proprietary product classification system has been developed, which products, nutrients and food characteristics are covered, and what are the details? | | [Information only, not to be scored] | |
|  | ***Policy position on engagement with government Reformulation policies*** | | | |
| **R-FORM9** | To what extent does the company engage with the government’s Healthy Food Partnership in relation to product reformulation, and in doing so support WHO’s position on product reformulation in relation to nutrients of concern?  As articulated in the Global Action Plan for the Prevention and Control of NCDs 2013-2020. | | 10: Fully engaged, representative on HFP working group and commit to signing on to action plans when developed  5: Somewhat engaged, through industry association/contributions to working groups/consideration of action plans set by the HFP  0: No engagement/ no information | |
|  | | | | |
| **R-LABEL** | **NUTRITION LABELLING** | | **MAXIMUM TOTAL POINTS = 55 (PRE-WEIGHTING)**  **WEIGHTING APPLIED = 15** | |
| **R-LABEL1.1** | Does the company commit to disclose nutrition information on its menus in states that do not mandate menu labelling? | | 10: Yes, relates to all menu items and commitment is publicly available  7.5: Yes, relates to all menu items, commitment is not publicly available  5: Yes, relates to some menu items and commitment is publicly available  2.5: Yes, relates to some menu items, commitment is not publicly available  0: No commitment / no information available to the research team | |
| **R-LABEL1.2** | What nutrition information does the company commit to providing on menus? | | *Up to 10 points maximum:*  5: Energy / calories  5: Symbol or logo indicating ‘healthy’ items according to overall nutritional profile  2: Sodium/salt  2: Saturated fat  2: Total fat  2: Trans fat  2: Sugar | |
| **R-LABEL2** | Does the company have a policy that they will provide comprehensive nutrition information in-store? | | 10: Yes, comprehensive nutrition information (kJ/calories, sodium, saturated fat, trans fat, total fat, sugar) for all regular menu items on a per 100g/100ml basis  7.5: Yes, comprehensive nutrition information (kJ/calories, sodium, saturated fat, trans fat, total fat, sugar) for all regular menu items  5: Comprehensive nutrition information for some regular menu items / commitment to provide some nutrition information in-store  2.5: Limited nutrition information for regular menu items  0: No policy / no information available to the research team | |
| **R-LABEL3** | Does the company have a policy to provide information on food composition to national authorities, on request?  (if applicable, e.g., information has been requested by government) | | 5: Yes, all products (published or not published)  2.5: Yes, some products  0: No policy / no information available to the research team | |
| **R-LABEL4** | Does the company provide nutrition information online? | | 10: Yes, comprehensive nutrition information (kJ/calories, sodium, saturated fat, trans fat, total fat, sugar) for all regular menu items, including on a per 100g/100ml basis  7.5: Yes, comprehensive nutrition information (kJ/calories, sodium, saturated fat, trans fat, total fat, sugar) for all regular menu items per serving only  5: Comprehensive nutrition information for some regular menu items  2.5: Limited nutrition information for regular menu items  0: No | |
|  | ***Policy position in relation to Menu labelling policies*** | | | |
| **R-LABEL5** | Does the company publish its position on preferred government policy with respect to menu labelling? | | 10: Yes, on own website  5: Yes, on industry association website  0: Not publicly available | |
|  | | | | |
| **R-PROMO** | **PROMOTION PRACTICES** | | **MAXIMUM TOTAL POINTS = 125 (PRE-WEIGHTING)**  **WEIGHTING APPLIED = 25** | |
| **R-PROMO1.1** | Does the company have an explicit policy to reduce the power and exposure of unhealthy food marketing to children on broadcast media (TV, radio)? | | 10: Yes, national policy and noted on company website / annual reports  7.5: Yes, global policy and noted on company website / annual reports  5: Yes, national policy but not noted on company website / annual reports  2.5: Yes, global policy but not noted on company website / annual reports  0: No policy / no information available to the research team | |
| **R-PROMO1.2** | To what age group(s) does the broadcast marketing policy apply? | | 10: 18 years and under  8: 16 years and under  6: 14 years and under  4: 12 years and under  2: Under 10 years  0: No policy / no information available to the research team | |
| **R-PROMO1.3** | How is the ‘target audience’ or ‘audience exposed’ defined? | | 10: Time-based restrictions, based on children’s peak viewing times (e.g., no advertising before 9:00pm  5: Based on audience share only including children (e.g. if >10% of total children are watching)  2.5: Based on audience share (e.g., if >10% of audience are children)  1: Children’s programmes only  0: No explicit threshold / definition available to the research team | |
| **R-PROMO2.1** | Does the company have an explicit policy to reduce the power and exposure of unhealthy food marketing to children on non-broadcast media (including websites, DVDs/games, social media, print media, product placement, outdoor marketing, in store marketing / point of sales marketing)? | | 10: Yes, national policy and noted on company website / annual reports  7.5: Yes, global policy and noted on company website / annual reports  5: Yes, national policy but not noted on company website / annual reports  2.5: Yes, global policy but not noted on company website / annual reports  0: No policy / no information available to the research team  [Information only – what specific media channels are included] | |
| **R-PROMO2.2** | To what age group(s) does the non-broadcast marketing policy apply? | | 10: 18 years and under  8: 16 years and under  6: 14 years and under  4: 12 years and under  2: Under 10 years  0: No policy / no information available to the research team | |
| **R-PROMO3** | Does the company commit not to sponsor children’s sporting, cultural or other activities using unhealthy products or brands? | | 10: Yes, comprehensive commitment including products and brands  5: Yes, comprehensive commitment including products only (brands still permitted)  2.5: Some commitments in the area, including some events or some forms of sponsorship  0: No commitment / no information available to the research team | |
| **R-PROMO4** | Does the company commit not to use marketing in settings where children gather using unhealthy brands (foods or company brands)? | | (Can be multiple)  2: Commits IN early childcare settings and primary schools (children up to age 11)  2: Commits NEAR (e.g. within 500m) of early childcare settings and primary schools (children up to age 11)  2: Commits IN secondary schools (children between age 12 and 18)  2: Commits NEAR (e.g., within 500m) of secondary schools (children between age 12 and 18)  2: Commits in other places where children gather (family and child clinics, paediatric services or other health facilities, sporting or recreation centres, or sporting or cultural events held at those premises) | |
| **R-PROMO5.1** | Does the company pledge not to use celebrities in marketing of products to children other than those that meet the company’s healthy standard? | | 5: All forms of marketing  2.5: Some forms of marketing (e.g., excludes packaging)  0: No policy / no information available to the research team | |
| **R-PROMO5.2** | Does the company pledge not to use fantasy and animation characters with a strong appeal to children in marketing of products other than those that meet the company’s healthy standard? | | 10: All forms of marketing (includes no use of characters with strong appeal to children across all forms of marketing)  5: Some forms of marketing (includes no use of characters with strong appeal to children across some forms of marketing)  2.5: Some restriction on use of fantasy/animation character in marketing (e.g. only applies to characters that appeal specifically to children)  0: No policy / no information available to the research team | |
| **R-PROMO5.3** | Does the company commit to not use premium offers (e.g., promotional toys, games, vouchers and competitions) in marketing of products (including as part of children’s meals) other than those that meet the company’s healthy standard? | | 5: All forms of marketing  2.5: Some forms of marketing (e.g., excludes packaging)  0: No commitment / no information available to the research team | |
| **R-PROMO6** | Does the company commit to only advertise or display ‘healthy’ sides and ‘healthy’ drinks in children’s combination meals in restaurants (for example, on menus and menu boards or in advertisements in restaurants)? | | 10: Yes, commits to only advertising both healthy sides and healthy drinks for children’s meals or does not advertise children’s meals  5: Yes, commits to only advertising either healthy sides or health drinks  0: No commitment / no information available to the research team | |
| **R-PROMO7** | Does the company audit its compliance with its policy on marketing to children at the national/country level? | | 5: Yes, the policy is audited by an independently appointed third party  2.5: Yes, the policy is independently audited  1: No, the policy is not independently audited  0: No auditing is conducted  DIVIDE POINTS BY HALF IF THE POLICY IS ONLY AUDITED AT THE GLOBAL LEVEL, AND NOT AT THE NATIONAL/COUNTRY LEVEL | |
| **R-PROMO8** | Does the company’s policy position support WHO’s position on government-led policy action related to reducing the exposure of children and adolescents to, and the power of, the marketing of unhealthy foods, as articulated in the WHO Global Action Plan for NCDs and other key WHO documents (such as the Report of the Commission on Ending Childhood Obesity)?  According to the World Health Assembly resolution WHA63.14, marketing policy should aim to reduce the impact on children of marketing of foods high in saturated fats, *trans-*fatty acids, free sugars or salt by reducing both exposure of children to, and power of, marketing of foods high in these nutrients, with uniform implementation across all implementing bodies. The policy should include settings where children gather. The government should be the key stakeholder in developing the policy including implementation, monitoring and evaluation, and enforcement systems should be in place including clear definitions of sanctions. | | 10: Strong support (e.g., includes support for government-led action of marketing to children and adolescents, related to power and exposure)  5: Weak support (e.g., includes support for government-led action of marketing to children or adolescents, related to either power or exposure,)  0: No details available  -5: Somewhat opposed (e.g., opposes government-led efforts to restrict some aspects of promotion to children / adolescents)  -10: Strongly opposed (e.g., opposes any actions to reduce promotion to children) | |
|  | Classification system | |  | |
| **R-PROMO9.1** | What system / criteria (e.g., product classification system or nutrient profiling system) does the company use to classify the healthiness of products for the purposes of promotion to children and adolescents? | | 10: Adopted official national or regional classification system (developed by WHO, PAHO, national government, etc.)  5: Developed own system that has been validated and shows strong alignment with official national (or regional) classification systems / dietary guidelines, published in peer-reviewed literature  2.5: Developed own system that has been validated and shows alignment with official national (or regional) classification systems / dietary guidelines, not published in peer-reviewed literature  0: No information / poor alignment / does not have a system | |
| **R-PROMO9.2** | If a proprietary product classification system has been developed, which products, nutrients and food characteristics are covered, and what are the details? | | [Information only, not to be scored] | |
|  | | | | |
| **R-ACCESS** | **PRODUCT ACCESSIBILITY** | | **MAXIMUM TOTAL POINTS = 95 (PRE-WEIGHTING)**  **WEIGHTING APPLIED = 20** | |
| **R-ACCESS1** | Does the company make a commitment to address the price and affordability of its healthy products relative to its unhealthy products? | | 10: Clear and specific targets for whole business, published and applied nationally  7.5: Clear and specific targets for whole business, not published and applied nationally  5: Broad commitment, published and applied nationally  2.5: Broad commitment, not published but disclosed to the research team  0: No commitment/ no policy information available to the research team | |
| **R-ACCESS2** | Does the company have a policy that price promotions are used only on healthy products?  (ONLY IF APPLICABLE) | | 10: Policy that all price promotions are for healthy products  5: Policy that factors the healthiness of products into price promotion decisions  2.5: No specific policy in the area, but ‘meal deals’ include the option of healthy sides and drinks  0: No commitment/ no policy information available to the research team | |
| **R-ACCESS3** | Does the company explicitly commit to not use price incentives (such as ‘supersizing’) that incentivise consumers to purchase larger portion sizes for minimal extra cost? | | 5: Yes  0: No explicit commitment / no policy information available to the research team | |
| **R-ACCESS4** | Does the company commit to not provide free refills for caloric soft drinks / soda?  (ONLY IF APPLICABLE) | | 5: Yes  0: No commitment/ no policy information available to the research team | |
|  | ***Policy position on Fiscal policies (WHO recommendations)*** | | | |
| **R-ACCESS5.1** | Does the company publish its policy position (in relation to government policy) on fiscal policies to make healthier foods relatively cheaper and unhealthy foods relatively more expensive? | | 10: Yes, on own website  5: Yes, on industry association website  0: Not publicly available | |
| **R-ACCESS5.2** | Does the company’s policy position support WHO’s position on fiscal policies to make healthier foods relative cheaper and unhealthy foods relatively more expensive, as articulated in the WHO Global Action Plan for NCDs and other key WHO documents (such as the Report of the Commission on Ending Childhood Obesity, Recommendation 1.2)?  (ECHO Statement on Recommendation 1.2: Implement an effective tax on sugar-sweetened beverages.)  (Global Action Plan: consider economic tools that are justified by evidence, and may include taxes and subsidies, that create incentives for behaviours associated with improved health outcomes, improve the affordability and encourage consumption of healthier food products and discourage the consumption of less healthy options.) | | 10: Strong support (e.g., includes support for taxes on unhealthy foods, broadly defined, as well as subsidies for healthy foods)  5: Weak support (e.g., includes support for taxes on unhealthy foods, narrowly defined, or subsidies for healthy foods)  0: No details available  -10: Opposed (e.g., opposes soft drinks tax/unhealthy foods tax OR subsidies on healthy foods) | |
| **R-ACCESS6** | Does the company commit to not opening new stores near schools? | | (Can be multiple)  5: Commits to not opening a new location near (e.g., within 500m) of primary schools, published (2.5 if not published)  5: Commits to not opening a new location near (e.g., within 500m) of secondary schools, published (2.5 if not published) | |
| **R-ACCESS7.1** | Does the company have a policy that ‘assigned’ or ‘default’ drink items included as part of children’s combination meals are healthy items (e.g., water)?  (ONLY IF APPLICABLE) | | 5: Yes, healthy drink items are assigned by default for all children’s combination meals  2.5: Yes, healthy drink items are assigned by default for some children’s combination meals  0: No policy/ no information available to the research team | |
| **R-ACCESS7.2** | Does the company have a policy that ‘assigned’ or ‘default’ drink items included as part of ‘non-children’s’ combination meals are healthy items (e.g., water)?  (ONLY IF APPLICABLE) | | 5: Yes, healthy drink items are assigned by default for all non-children’s combination meals  0: No policy/ no information available to the research team | |
| **R-ACCESS8.1** | Does the company have a policy that ‘assigned’ or ‘default’ side items included as part of children’s combination meals are healthy items (e.g., salad, fruit, vegetables)?  (ONLY IF APPLICABLE) | | 5: Yes, for all children’s meals  2.5: if children’s meals offer ‘healthier’ sides as one of the options, but not the default)  0: No policy/ no information available to the research team | |
| **R-ACCESS8.2** | Does the company have a policy that ‘assigned’ or ‘default’ side items included as part of ‘non-children’s’ combination meals are healthy items (e.g., salad, vegetables)?  (ONLY IF APPLICABLE) | | 5: Yes, for other meals (non-children’s meals)  2.5: if other meals offer ‘healthier’ as one of the options, but not the default  0: No policy/ no information available to the research team | |
|  | ***Classification system*** | |  | |
| **R-ACCESS9.1** | What system / criteria (e.g., product classification system or nutrient profiling system) does the company use to classify the healthiness of products for the purposes of product accessibility (e.g. pricing, availability and selection)? | | 10: Adopted official national or regional classification system (developed by WHO, PAHO, national government, etc.)  5: Developed own system that has been validated and shows strong alignment with official national (or regional) classification systems / dietary guidelines, published in peer-reviewed literature  2.5: Developed own system that has been validated and shows alignment with official national (or regional) classification systems / dietary guidelines, not published in peer-reviewed literature  0: No information / poor alignment / does not have a system | |
| **R-ACCESS9.2** | If a proprietary product classification system has been developed, which products, nutrients and food characteristics are covered, and what are the details? | | [Information only, not to be scored] | |
|  | | | | |
| **R-RELAT** | **RELATIONSHIPS WITH EXTERNAL GROUPS** | | **MAXIMUM TOTAL POINTS = 80 (PRE-WEIGHTING)**  **WEIGHTING APPLIED = 5** | |
| **R-RELAT1** | Does the company publish details of the professional organisations (e.g., professional associations for nutrition or dietetics, physical activity or exercise organisations, medical organisations or societies, etc.) and/or scientific events (e.g., conferences) it funds or supports, including awards/prizes, making clear the nature of that support? | | 10: Yes, information on national-level activity is publicly available (website or document) in a consolidated and cumulative form  5: Yes, information is available, but is not consolidated and easy to locate OR information is available at the global level only OR comprehensive information about their activities in the area provided to the project team  0: No information available / provided  n/a: Active declaration stating no activity in this area (either publicly available or disclosed to research team) | |
| **R-RELAT2** | Does the company publish details of the external research (e.g., conducted by individuals/groups/organisations) it funds or supports, including awards/prizes? | | 10: Yes, information on national-level activity is publicly available (website or document) in a consolidated and cumulative form  5: Yes, information is available, but is not consolidated and easy to locate OR information is available at the global level only OR comprehensive information about their activities in the area provided to the project team  0: No information available / provided  n/a: Active declaration stating no activity in this area (either publicly available or disclosed to research team) | |
| **R-RELAT3** | For philanthropic funding, does the company publish details of the groups or organisations it funds or supports? | | 10: Yes, information on national-level activity is publicly available (website or document) in a consolidated and cumulative form  5: Yes, information is available, but is not consolidated/comprehensive and easy to locate OR information is available at the global level only OR comprehensive information about their activities in the area provided to the project team  0: No information available / provided  n/a: Active declaration stating no activity in this area (either publicly available or disclosed to research team) | |
| **R-RELAT4.1** | Does the company publish details of the nutrition education / healthy diet oriented programs it funds or supports? | | 10: Yes, information on national-level activity is publicly available (website or document) in a consolidated and cumulative form  5: Yes, information is available, but is not consolidated and easy to locate OR information is available at the global level only OR comprehensive information about their activities in the area provided to the project team  0: No information available / provided  n/a: Active declaration stating no activity in this area (either publicly available or disclosed to research team) | |
| **R-RELAT4.2** | For nutrition education / health diet oriented programs, does the company have a commitment to align programs to national or regional dietary guidelines? | | [Information only, not to be scored] | |
| **R-RELAT5** | Does the company publish details of the active lifestyle programs (sports, physical activity) it funds or supports? | | 10: Yes, information on national-level activity is publicly available (website or document) in a consolidated and cumulative form  5: Yes, information is available, but is not consolidated and easy to locate OR information is available at the global level only OR comprehensive information about their activities in the area provided to the project team  0: No information available / provided  n/a: Active declaration stating no activity in this area (either publicly available or disclosed to research team) | |
| **R-RELAT6** | Does the company publish details of its involvement in public-private partnerships and/or joint ventures with government organisations / agencies? (in addition to those covered as part of R-RELAT4.1 and R-RELAT5) | | 10: Yes, information on national-level activity is publicly available (website or document) in a consolidated and cumulative form  5: Yes, information is available, but is not consolidated and easy to locate OR information is available at the global level only OR comprehensive information about their activities in the area provided to the project team  0: No information available / provided  n/a: Active declaration stating no activity in this area (either publicly available or disclosed to research team) | |
| **R-RELAT7** | Does the company publish details of its political donations? | | 10: Yes, information on national-level activity is publicly available (on a company website or document) OR active declaration of no activity in this area (either publicly available or disclosed to research team)  0: No information available / provided | |
| **R-RELAT8** | Does the company publish its membership / support for / ownership of industry associations, think tanks, interest groups, community organisations or other organisations that lobby in relation to population nutrition and/or obesity and NCD issues? | | 10: Yes, information on national-level activity is publicly available (website or document) in a consolidated and cumulative form  5: Yes, information is available, but is not consolidated and easy to locate OR information is available at the global level only OR comprehensive information about their activities in the area provided to the project team  0: No information available / provided  n/a: Active declaration stating no activity in this area (either publicly available or disclosed to research team) | |
| **R-RELAT9** | Does the company have written policy and guidelines related to any of the above (funding or support for professional organisations, external research, philanthropic funding, nutrition education / healthy diet oriented programs, active lifestyle programs), including details of how it will be involved in these activities? | | [Information only, not to be scored] | |
|  | |  | |  |
