# Supplementary material for: Benchmarking the Nutrition-Related Policies and Commitments of Major Food Companies in Australia, 2018
Source: Int J Environ Res Public Health. 2020 Aug 22;17(17):6118. doi: 10.3390/ijerph17176118 (PMC7504100; doi:10.3390/ijerph17176118)
Supplement: Supplementary file 1 [file ijerph-17-06118-s001.zip › Table S4 - BIA-Obesity Australia 2018 IJERPH.docx]

Supplementary material

Table S4: Good practice examples by domain

| **Domain** | **Selected good practice examples ^[[1]](#footnote-1)^** |
| --- | --- |
| **Corporate strategy** | 1. *Unilever* has made a strong commitment to nutrition and health at the global level, and highlights health as one of three key pillars that make up the company’s Sustainable Living Plan. It sets out specific, time-bound nutrition and health targets and objectives that are routinely reported against. It aligns its approach with priorities set out by the World Health Organization (WHO) and the United Nations (UN) Sustainable Development Goals (1). 2. *Nestlé* and *Lion Dairy and Drinks* (Australia) publish annual national reports detailing their progress against their objectives and targets related to nutrition, and demonstrate that Key Performance Indicators (KPI) of senior managers are linked to their nutrition strategy. |
| **Product formulation** | 1. *Nestlé* has set specific, time-bound reformulation targets for the reduction of saturated fat, salt and added sugar across the company’s global portfolio, and has reported removal of all artificially produced trans fats in its products. Compliance with reformulation targets is audited and publicly disclosed on a regular basis (2). 2. Supermarket chain *Foodstuffs New Zealand (NZ)* have committed to reformulating own-brand products to support the NZ government’s 2015 Childhood Obesity plan. The company had set a target to reduce sodium and sugar by 10% by the end of 2018 across their home brand product range and pledge to continually reduce saturated fat and encourage reformulation of supplier products (3). 3. *McDonald’s* had made a global commitment to reduce the salt, sugar and fat of its product portfolio and to serve 100% more fruit, vegetables, low fat dairy or wholegrains by 2020. They committed to offering healthier drinks and side items, like fruit, water and low-fat dairy, for children’s meals (4). |
| **Nutrition labelling** | 1. *Sanitarium* (Australia) has a commitment to implement the Australian government-endorsed Health Star Rating system across all products in its portfolio, and provides comprehensive online nutrition information for all products (per serve, per 100g and HSR) (5). 2. *Woolworths* and *Coles* (Australia) have committed to implement the Australian government-endorsed HSR system across all own-brand products, including confectionery products (6). 3. *Walt Disney (US)* restaurants, resorts and theme parks have introduced an icon to easily identify healthier menu items (in-store and online) (7). |
| **Promotion practices** | 1. *Danone* implements a marketing to children policy (applying to children under 12 years) that covers broadcast and non-broadcast media. It commits to not use promotional techniques that appeal to children, and does not market in the vicinity of schools, in secondary schools, or in places where children gather (e.g., day care centres, activity centres). It makes a global commitment to not use messaging that encourages a ‘less healthy’ lifestyle or overconsumption of any of its products. *Danone* reports compliance with its policies, and discloses and audits its marketing spending on healthy products, with a view to increasing marketing spending on these products over time (8). 2. *Woolworths* (Australia) has an ongoing ‘free fruit for kids’ initiative, providing free fresh fruit to children under 12 in most stores nationally (9). 3. Several major quick service restaurant chains have made a commitment to not offer toys in children’s meals, e.g., *Jack in the Box* (US), *KFC Australia* (10). |
| **Product accessibility** | 1. *Unilever* has set a clear, time-bound target to transition a portion of its portfolio into a healthier product category. The company makes a commitment to increase the affordability and availability of ‘healthy’ products across its global markets and identifies accessibility issues as a key part of its business strategy, aligning with the UN’s Sustainable Development Goals (1). 2. *Tesco United Kingdom (UK)* removed all sweets and chocolates from its checkouts, including its Express and Metro stores. Confectionery-free checkouts were implemented across the UK from 2015 and replaced with healthy items, such as dried fruits and nuts (11). 3. *Subway* in Australia and the United States (US) provide a healthier side and drink option as the default in its children’s meals, and in the US explicitly aligns children’s meals (including side and drink items) with US Department of Agriculture (USDA) Dietary Guidelines for Americans. |
| **Relationships with external groups** | 1. *Coca-Cola International* publishes a list of the external groups it funds/supports, including details of the nature, date and amount of support/funding given to research institutions, health professionals, scientific experts, professional associations and partnerships related to health and nutrition. They disclose all nutrition and active lifestyle programs with which they are affiliated, and all information is updated annually (12). 2. Several companies, including *Nestlé*, *Unilever*, *Campbell Arnott’s,* *Mars* and *George Weston Foods*, have formal policies at the global level that prohibit political donations. |

References

1. Unilever. Improving Nutrition. 2018 [Updated 2020; cited 24 Jul 2020]. Available from: <https://www.unilever.com/sustainable-living/the-sustainable-living-plan/improving-health-and-well-being/improving-nutrition/>.

2. Nestlé. Reducing sugars, sodium and fat. 2018 [Updated 2020; cited 24 Jul 2020]. Available from: <https://www.nestle.com/csv/impact/tastier-healthier/sugar-salt-fat>.

3. Foodstuffs. Product Reformulation. 2018 [Updated 2020; Cited 24 Jul 2020]. Available from: <https://www.foodstuffs.co.nz/corporate-responsibility/foodstuffs%E2%80%99-pledge-to-promote-healthier-eating/product-reformulation/>.

4. McDonald's. The Good Business Report (2014). McDonald's; 2015.

5. Sanitarium. What is the Health Star Rating on food packaging? 2018 [Updated 2020; cited 24 Jul 2020]. Available from: <https://www.sanitarium.com.au/food-nutrition/nutrition/health-star-rating>.

6. Woolworths. Healthy Eating. 2018 [Updated 2020; cited 24 Jul 2020]. Available from: <https://www.woolworths.com.au/Shop/Discover/healthy-eating>

7. The Disney Food Blog. Mickey Check Meals. 2016 [cited 24 Jul 2020]. Available from: <https://www.disneyfoodblog.com/tag/disney-nutrition-guidelines/>.

8. Danone. Pledge on advertising aimed at children. 2013 [cited 24 Jul 2020]. Available from: <https://www.danone.com/content/dam/danone-corp/danone-com/about-us-impact/policies-and-commitments/en/2013/2013_08_PledgeOnAdvertisingAimedAtChildren.pdf>.

9. Woolworths Group. Free Fruit for Kids at Woolworths. 2015 [cited 24 Jul 2020]. Available from: <https://www.woolworthsgroup.com.au/page/media/Latest_News/Free_Fruit_for_Kids_at_Woolworths/>.

10. AdAge. Jack in the Box eliminates toys from kids meals. 2011 [cited 24 Jul 2020]. Available from: <https://adage.com/article/news/jack-box-eliminates-toys-kids-meals/228334>.

11. Tesco. Sweets and chocolates removed from all checkouts from today. 2015 [cited 24 Jul 2020]. Available from: <https://www.tescoplc.com/news/2015/sweets-and-chocolates-removed-from-all-checkouts-from-today/>.

12. The Coca-Cola Company. Transparency Research Reports. 2018 [cited 24 Jul 2020]. Available from: <https://www.coca-colacompany.com/policies-and-practices>.

1. As at 2018 [↑](#footnote-ref-1)
